# Supplementary material for: Implications of reducing antibiotic treatment duration for antimicrobial resistance in hospital settings: A modelling study and meta-analysis
Source: PLoS Med. 2023 Jun 15;20(6):e1004013. doi: 10.1371/journal.pmed.1004013 (PMC10270346; doi:10.1371/journal.pmed.1004013)
Supplement: S1 Text — Table A. Parameters used in the models and their respective ranges obtained from literature review. (DOCX) [file pmed.1004013.s001.docx]

**Supplementary material 1: Model details and exploration**

**S1.1 Model details**

The agent-based models were set up to simulate a randomised controlled trial involving two identical hospital wards. Patients who were admitted to the hospital were randomly assigned to either wards. These two wards shared similar ward and patient characteristics i.e. lengths of stay, bed capacity, transmission rate of resistant bacteria, proportion of patients who required antibiotics treatment and proportion of patients who were resistance carriers at admission. The wards were assumed to be fully occupied at all times.

Patients were prescribed antibiotics for clinical infections at admission and during hospitalisation. The prescribed antibiotics could be active against both susceptible and resistant bacteria or active against only susceptible bacteria. The wards differed only in the antibiotic duration. For example, in the scenario where antibiotics administered were active against both susceptible and resistant bacteria, patients admitted to the long duration ward who required antibiotics received this type of antibiotic for a long duration while the patients in the short duration warded received the same type of antibiotic for a shorter duration.

The patients' colonisation status were updated daily, depending on if antibiotics were prescribed, if the antibiotics prescribed were active against resistant bacteria or selectively promoting the growth of resistant bacteria, transmission of antibiotic resistant bacteria and bacterial growth (main text Fig 1). The time step was kept sufficiently small at one day intervals such that the sum of probabilities for competing events were less than one. This allowed for only one event to take place during each time step.

A new resistance carrier may be added to the ward in three ways: i) transmission events, i.e., newly acquiring resistant bacteria from existing resistance carriers in the ward, ii) within-host selection of pre-existing resistant bacteria due to receiving antibiotics which are only active against susceptible bacteria, iii) resistance carriers are admitted to the ward. The probability of being transmitted resistant bacteria depended on the proportion of patients in the ward who were existing resistance carriers:

*ζ_i,t_* ∼ *Bernoulli*${(1 - (1-\varphi)}^{\rho_{r.bed, t}})$

where *ζ_i,t_* is a binary indicator which takes the value of 1 if a transmission event was to occur and 0 otherwise, for an individual *i* at time step *t*; $\varphi$ is the probability for a transmission event to occur; *ρ_r.bed,t_* is the proportion of patients in the ward who were resistant carriers at time step *t*.

The three models are described in detail below.

- Exclusive colonisation model

Patients may assume any of the three carriage status: high proportion of susceptible bacteria (*S*), low proportion of susceptible bacteria (*s*), or multidrug resistant bacteria (*R*). Co-carriage of susceptible and resistant bacteria was not allowed in this model.

When on administered antibiotics which were active against both susceptible and resistant bacteria, an individual with *S* or *R* state might change to s in the next time step due to effective antibiotic killing. Without antibiotics, an individual in *s* state might change to *S* due to regrowth of the susceptible bacteria, and an individual in *R* state might decolonise spontaneously to *S*. An individual in either *s* or *R* states became a resistant carrier if there was a transmission event. Individuals in state *S*, by having a high proportion of susceptible bacteria, were protected from acquiring drug resistant bacteria though the ‘bacterial interference factor’, *b*: [3]

$$\varphi_{S\to R} = \varphi_{s\to R} (1 - b)$$

- Co-colonisation model

Co-carriage of susceptible and resistant bacteria was incorporated in this model. Patients might be in any of the five carriage status: high proportion of susceptible bacteria (*S*), low proportion of susceptible bacteria (*s*), dominant population of susceptible bacteria with some resistant bacteria (*Sr*), low proportion of both susceptible and resistant bacteria (*sr*) and dominant population of resistant bacteria with some susceptible bacteria (*sR*).

When on effective antibiotics, an individual with *S*, *Sr* or *sR* state might change to *s* or *sr* due to antibiotic killing. However when the administered antibiotics were only active against susceptible bacteria, selective pressure might promote the growth of resistant bacteria and enable an individual to change from *sr* to *sR*. Without antibiotics, susceptible and resistant bacteria might grow and change *s* or *sr* to *S* or *Sr* states respectively, or *sr* to *sR*.

Spontaneous decolonisation might also occur in the absence of antibiotics, resulting in individuals to change from *sr* or *Sr* states to *s* or *S* respectively. Transmission events for individuals in *S* or *s* states might change their status to *Sr* or *sr* respectively. Only patients who carried a high proportion of resistant bacteria (*sR*) could transmit resistance to others and were considered resistance carriers. Similar to the exclusive colonisation model, individuals in *S* state were protected from acquiring drug resistant bacteria through the bacterial interference factor.

- Within-host growth model

The within-host growth model explicitly calculated the amounts of susceptible and resistant bacteria with the logistic growth function, which describes the typical bacterial population growth in lag, exponential growth and stationary phases. The exponential bacterial growth rate was proportional to the existing population size and decreased as the population size approaches the maximum carrying capacity, imposed by limited resources in the environment. We assumed that both susceptible and resistant bacteria occupied a similar ecological niche and hence shared the same maximum carrying capacity. The daily amounts bacteria were a sum of bacterial growth, transmitted bacteria in the receiving host and antibiotic killing.

In each iteration, a newly-admitted individual, *i*, has a total carrying capacity for bacteria of $e^{k_{i}}$/ml, where *k_i_* ∼ *N*(*µ_k_,*1). The amount of resistant bacteria, *N_i_^r^* carried by the individual, *i*, at the time of admission, *t* = 0, is $N_{i, t = 0}^{r}$ = *ρ_e_*$e^{k_{i}}$*ρ_r,i_*, where *ρ_e_* is the proportion of the total carrying capacity of bacteria present, and *ρ_r,i_* is the proportion of the existing amount of bacteria present that is resistant and *ρ_r,i_* ∼ *Beta*(0*.*2*,* 2). The amount of susceptible bacteria, *N_i_^s^* carried by the individual, *i*, at the time of admission, *t* = 0, is $N_{i, t = 0}^{s}$= ${\rho_{e}e}^{k_{i}}$ *−* $N_{i, t = 0}^{r}$.

The change in the amount of susceptible bacteria after admission, *i*, per time step, is a sum of the following processes,

1. Growth is modelled with the logistic growth function

$$G_{i,t}^{s} = c^{s}N_{i, t-1}^{s}(1 - \frac{N_{i, t-1}^{s}}{e^{k_{i}}})$$

where $c^{s}$is the growth constant for susceptible bacteria.

1. Antibiotic killing (density-dependent)

$A_{i, t}^{s} = \delta_{i, t-1} \alpha^{s}{(N}_{i, t-1}^{s} + G_{i, t}^{s})$

where *δ_i,t_* takes a value of 1 if the individual *i* was on antibiotics at time step *t*−1 and 0 otherwise, and $\alpha^{s}$ is the antibiotic killing constant.

Hence, the rate of change in susceptible bacteria within an individual, *i*, per time step, *t*, is,

$\Delta N_{i,t}^{s} = G_{i,t}^{s} - A_{i,t}^{s}$ .

After admission, the change in the amount of resistant bacteria for an individual, *i*, per time step, *t*, is a sum of the following processes,

1. Growth is modelled with the logistic growth function

$$G_{i,t}^{r} = c^{r}N_{i, t-1}^{r}(1 - \frac{N_{i, t-1}^{s} + N_{i, t-1}^{r}}{e^{k_{i}}})$$

*c^r^* = *fc^s^*

where *c^r^* is related to *c^s^* by *f*, a fitness factor for resistant bacteria.

1. Antibiotic killing (density-dependent)

$$A_{i,t}^{r} = \delta_{i, t-1}^{'}\alpha^{r}{(N}_{i, t-1}^{r} + G_{i,t}^{r})$$

where *α^r^* is the antibiotic killing constant and $\delta_{i, t-1}^{'}$takes a value of 1 if the individual, *i,* was on antibiotics at time step, *t* −1, and 0 otherwise.

1. Transmission

$$T_{i,t}^{r} = \zeta_{i, t}\tau N_{i,t}^{r}$$

where *ζ_i,t_* is a binary indicator which takes the value of 1 if a transmission event was to occur and 0 otherwise for an individual, *i,* at time step, *t*, and $\tau N_{i,t}^{r}$is the amount of resistant bacteria transmitted to the receiving individual, *i*.

Hence, the rate of change in resistant bacteria within an individual, *i*, per time step, *t*, is,

$\Delta N_{i,t}^{r} = G_{i,t}^{r} - A_{i,t}^{r} + T_{i,t}^{r}$.

When$N_{i,t}^{s} + N_{i,t}^{r} > e^{k_{i}}$, we set $N_{i,t}^{r} = \frac{N_{i,t}^{r}}{N_{i,t}^{s} + N_{i,t}^{r}} e^{k_{i}}$ and $N_{i,t}^{s} = \frac{N_{i,t}^{s}}{N_{i,t}^{s}} e^{k_{i}}$ i.e. ensuring that the total carrying capacity could not be exceeded.

An individual carrying an amount of resistant bacteria higher than the threshold, *N^t^*, is considered as resistance carrier. *N^t^* is a proportion of the existing amount of bacteria and is given by *N^t^* = Γ*ρ_e_e^k^*.

**Table A. Parameters used in the models and their respective ranges obtained from literature review**

| **Symbol** | **Description** | **Distribution** | **Models** | **Range** | **Unit** | **Reference^** |
| --- | --- | --- | --- | --- | --- | --- |
| **Ward level** | | | | | | |
| *n* | Number of beds in the ward | Uniform | 1, 2, 3 | 5-50 | Beds |  |
| *l* | Maximum length of stay for each patient in the ward | Exponential | 1, 2, 3 | 3-20 | Days |  |
| **Individual level** | | | | | | |
| **Carriage status for each patient on the first day of admission to the ward** | | | | | | |
| *p_R_^~^* | Probability of carrying resistant bacteria (*R*) | Uniform | 1, 2, 3 | 0.01-0.8 |  |  |
| *p_r_^~^* | Probability of carrying low proportion of resistant bacteria (*Sr* or *sr*) | Uniform | 2 | 0-1 |  |  |
| *p_Sr_^~^* | Probability of carrying high proportion of susceptible and low proportion of resistant bacteria (*Sr*) | Uniform | 2 | 0-1 |  |  |
| *p_S_^~^* | Probability of carrying susceptible bacteria (*S*) | Uniform | 1, 2 | 0-1 |  |  |
| $\rho_{e}$ | Proportion of the total carrying capacity occupied by bacteria | Normal | 3 | 0.1-0.9 |  | [4,5] |
| K | Mean total carrying capacity for bacteria (log scale) | Normal | 3 | $e^{18 - 24}$ | Bacteria/ml | [6] |
| **Growth of bacteria** | | | | | | |
| *g_s_* | Probability of susceptible bacteria repopulating (*s* 🡪 *S* and *sr* 🡪 *Sr*) | Uniform | 1, 2 | 0.02-0.12 |  | [7–9] |
| *c_s_* | Exponential growth constant for susceptible bacteria | Uniform | 3 | 0.1-2 |  | [10] |
| *f* | Growth fitness factor for resistant bacteria (multiplied to *c_s_* to derive exponential growth constant for resistant bacteria) | Uniform | 2, 3 | 0.5-2 |  | [11] |
| **Transmission of resistant bacteria** | | | | | | |
| *Γ* | The threshold proportion of resistant bacteria carried for the carrier to transmit resistant bacteria to others | Uniform | 3 | 0.01-0.2 |  |  |
| *τ* | Proportion of resistant bacteria carried that is transmitted during a transmission event | Uniform | 3 | 0.01-0.5 |  |  |
| $\varphi$ | Probability of transmitting resistant bacteria from resistance carrier to non-resistance carriers | Uniform | 1, 2, 3 | 0.001-0.3 |  | [12,13] |
| *b^~^* | Bacterial interference factor, a protection factor against being transmitted resistant bacteria in those carrying a high proportion of susceptible bacteria compared to those carrying a low proportion of susceptible bacteria on a relative scale | Uniform | 1, 2 | 0-1 |  |  |
| **Decolonisation of resistant bacteria** | | | | | | |
| $\mu$ | Probability of resistance decolonisation (*R* 🡪 *S*) | Uniform | 1 | 0.002-0.02 |  | [14] |
|  | Probability of resistance decolonisation (*sR* 🡪 *sr*, *Sr* 🡪 *S* and *sr* 🡪 *s*) | Uniform | 2 | 0.002-0.02 |  |  |
| **Antibiotic killing** | | | | | | |
| $\alpha_{s}$*^~^* | Probability of bacterial killing by an antibiotic active against susceptible bacteria (*S* and *s*) | Uniform | 1, 2 | 0.1-0.5 |  | [15–17] |
|  | Mean proportion reduction in susceptible bacteria due to effective antibiotic killing | Uniform | 3 | 0.1-1 |  |  |
| $\alpha_{r}$*^~^* | Probability of bacterial killing by an antibiotic active against resistant bacteria (*R* and *r*) | Uniform | 1, 2 | 0.1-0.5 |  |  |
|  | Mean proportion reduction in resistant bacteria due to effective antibiotic killing | Uniform | 3 | 0.1-1 |  |  |
| **Number of antibiotic prescriptions** | | | | | |  |
| $\omega_{day1}$*^~^* | Probability of being prescribed any antibiotics on admission day one | Uniform | 1, 2, 3 | 0.1-1 |  |  |
| $\omega_{day1.r}$*^~^* | Probability of the antibiotic prescribed on admission day one is active against resistant bacteria | Uniform | 1, 2, 3 | 0.1-1 |  |  |
| $\omega_{after}$*^~^* | Probability of being prescribed any antibiotics during admission | Unform | 1, 2, 3 | 0.001 – 0.15 |  |  |
| $\omega_{after.r}$*^~^* | Probability of the antibiotic prescribed during admission is active against resistant bacteria | Uniform | 1, 2, 3 | 0.1-1 |  |  |

^ References are provided for parameters values which could be found in the literature. For those parameter values not found in the literature, we explored the largest reasonable range of parameter values.

*^~^* Parameter ranges in terms of proportions or daily probabilities which are not supported by references were chosen using the largest sensible ranges.

**S1.3 Model exploration**

We used parameter ranges found in the literature (Table A in S1 Text) and performed global sensitivity analysis using Latin Hypercube sampling and Partial Rank Correlation Coefficient (LHS-PRCC). LHS, a Monte Carlo sampling method, is favoured for its computing efficiency.[18] PRCC is a highly efficient and reliable measure that accounts for non-linear but monotonic relationships between the parameters and model output.[19] We calculated the confidence intervals for the PRCCs using bootstrapping (1000 times).

Prior to calculating PRCC, we examined the correlations of each parameter with the model output for non-monotonicities via visualisation of scatter plots, Spearman’s rank correlation measure and Hoeffding’s D measure.[20,21] The optimal sample size for each sensitivity analyses was chosen when additional parameter combinations did not alter the output of the sensitivity analysis significantly. This is calculated using the concordance measure, Symmetrised Blest Measure of Association (SBMA) to confirm the optimal number of parameter combinations).[22]

S1.3.1 Optimising number of iterations

Because the models are stochastic, i.e. each decision step is based on a probability and every iteration will not produce the same decision outcome even when repeated with the same probability inputs, the inherent randomness reduces the efficiency of sensitivity analysis. To minimise this, we discarded a proportion of the initial runs to ensure equilibrium of the model is reached prior to calculating the model outputs (Fig A in S1 Text).


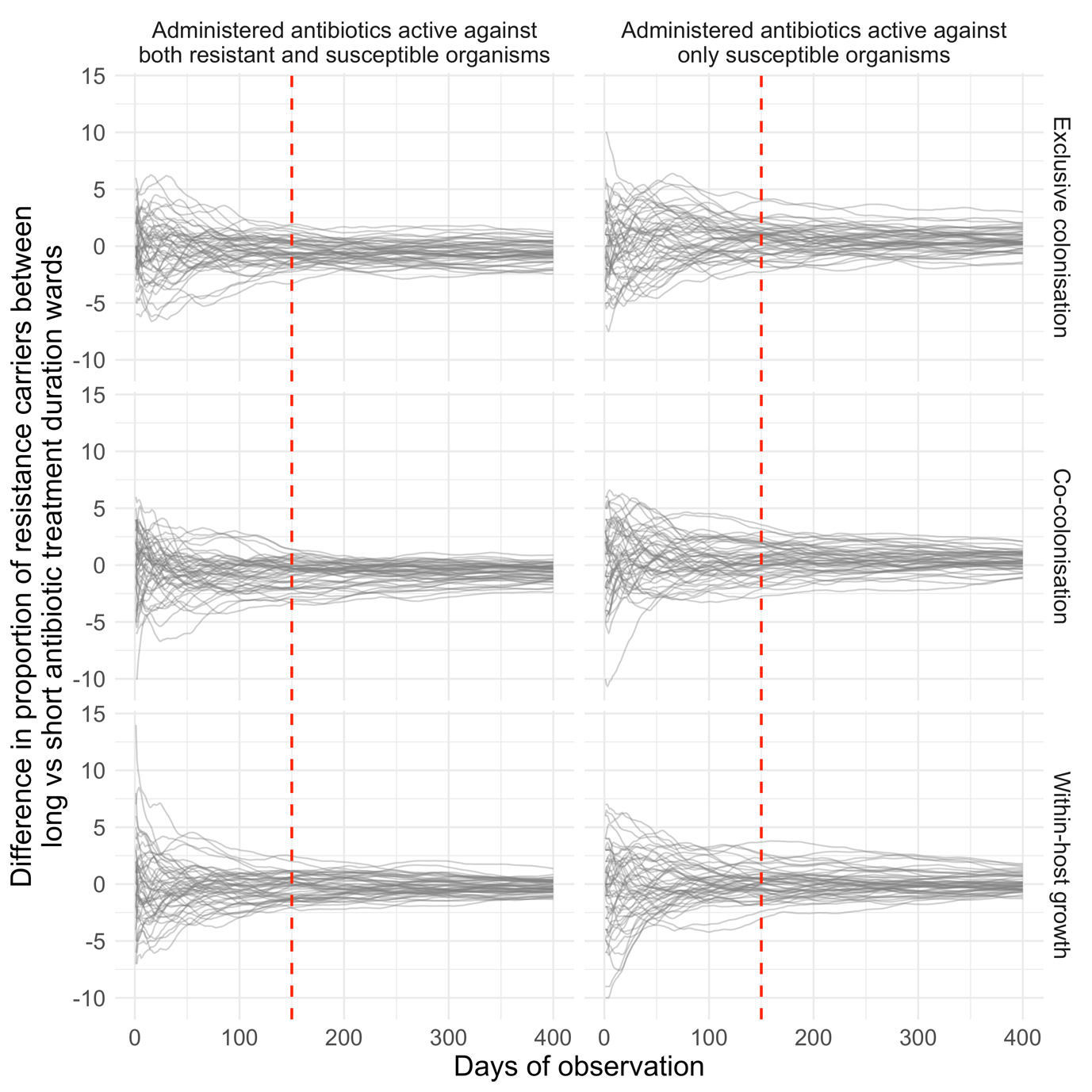


Fig A. Checking equilibrium state of the models. To find the number of days taken for the models to reach equilibrium, we plotted the output, absolute difference in the number of resistance carriers per day, against the number of days of observation. The intervention here was varying the duration of antibiotic treatment between the two wards. Each grey line represents the output from one iteration. The same set of parameter values was used for each iteration. Equilibrium states were generally reached after 150 days for all models (red dashed line). Hence in the simulations, the models are ran over 300 days with the first 150 days discarded.

In addition, we averaged the model outputs from a number of iterations for the same set of parameter combination inputs, which has been shown to improve reproducibility at smaller sample sizes and strengthen correlation between parameter values and model output during sensitivity analyses.[19] To find the optimal number of iterations per simulation, we compared distributions of simulation outputs under identical parameter values using the Vargha-Delaney A-Test, which is a nonparametric measure of the difference between the distributions of model outputs suggesting if the outputs are consistent (Fig B in S1 Text).[23,24]


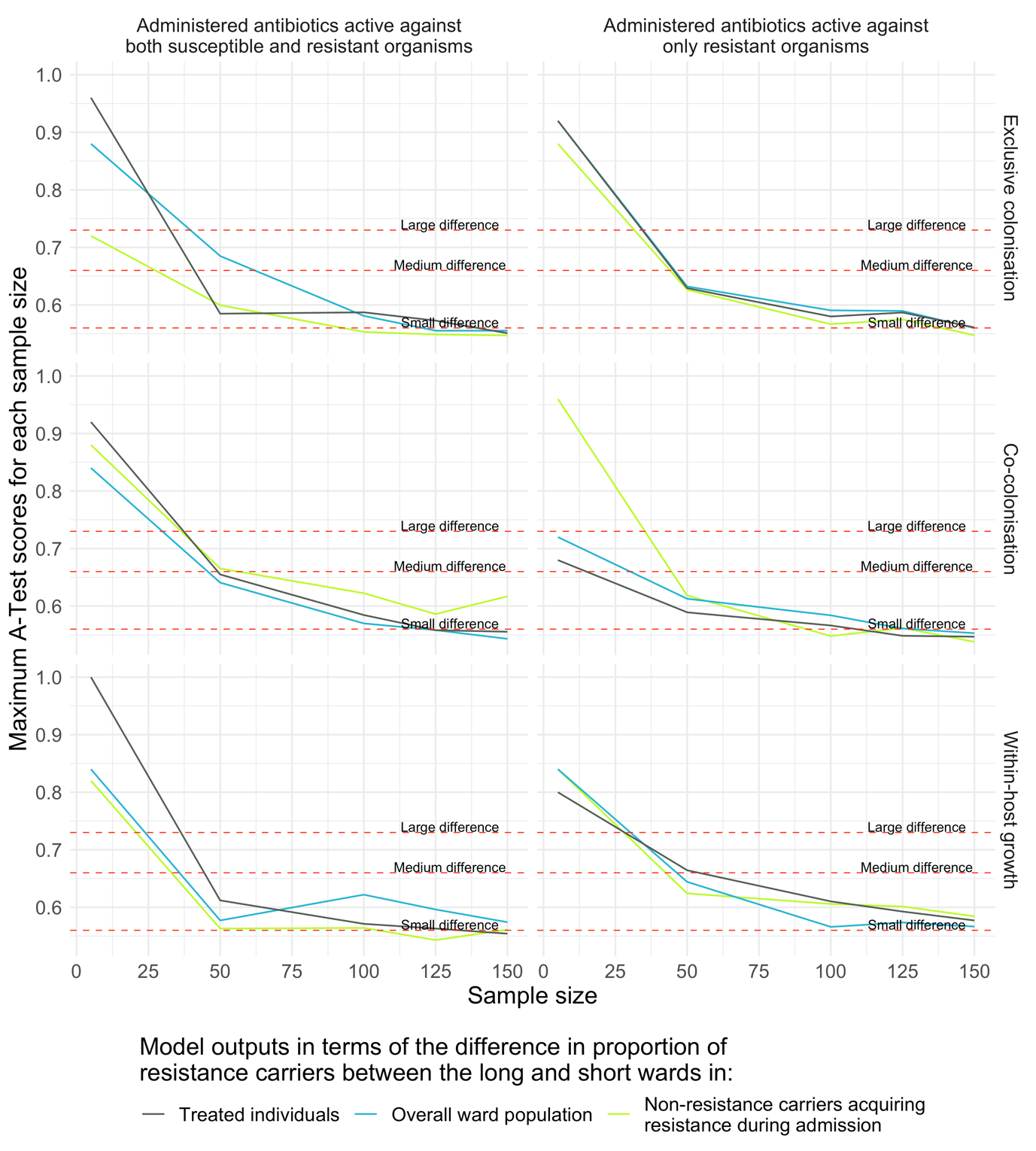


Fig B. Determining optimal number of iterations for each model. Consistency analysis was performed with the Vargha-Delaney A-Test to find the optimal number of iterations for each simulation. The horizontal dashed lines represent arbitrary A-Test score thresholds for small, medium and large differences at 0.56, 0.66 and 0.73 respectively. The graphs on the left panels are the results from the scenario where the resistance mechanism is third generation cephalosporin resistance; the graphs on the right panels are those from the scenario where the resistance mechanism is carbapenem resistance. We used 100 iterations per set of parameters values for all simulations.

S1.3.2 Correlation measures to ensure monotonicity

Non-monotonicities in the correlations between the parameters and the model output were examined visually through scatter plots and calculating the Hoeffding's D measure and Spearman's rank correlation measure. There were no non-monotonicities found within the explored parameter space.

- Scatter plots

The following scatter plots display the model outputs as a function of each parameter. The parameter values are sampled from the hypercubes formed within each parameter space. 500 unique sets of parameters were explored in each parameter space. A limitation here is that not all combinations of parameters could be explored.

1. Exclusive colonisation model


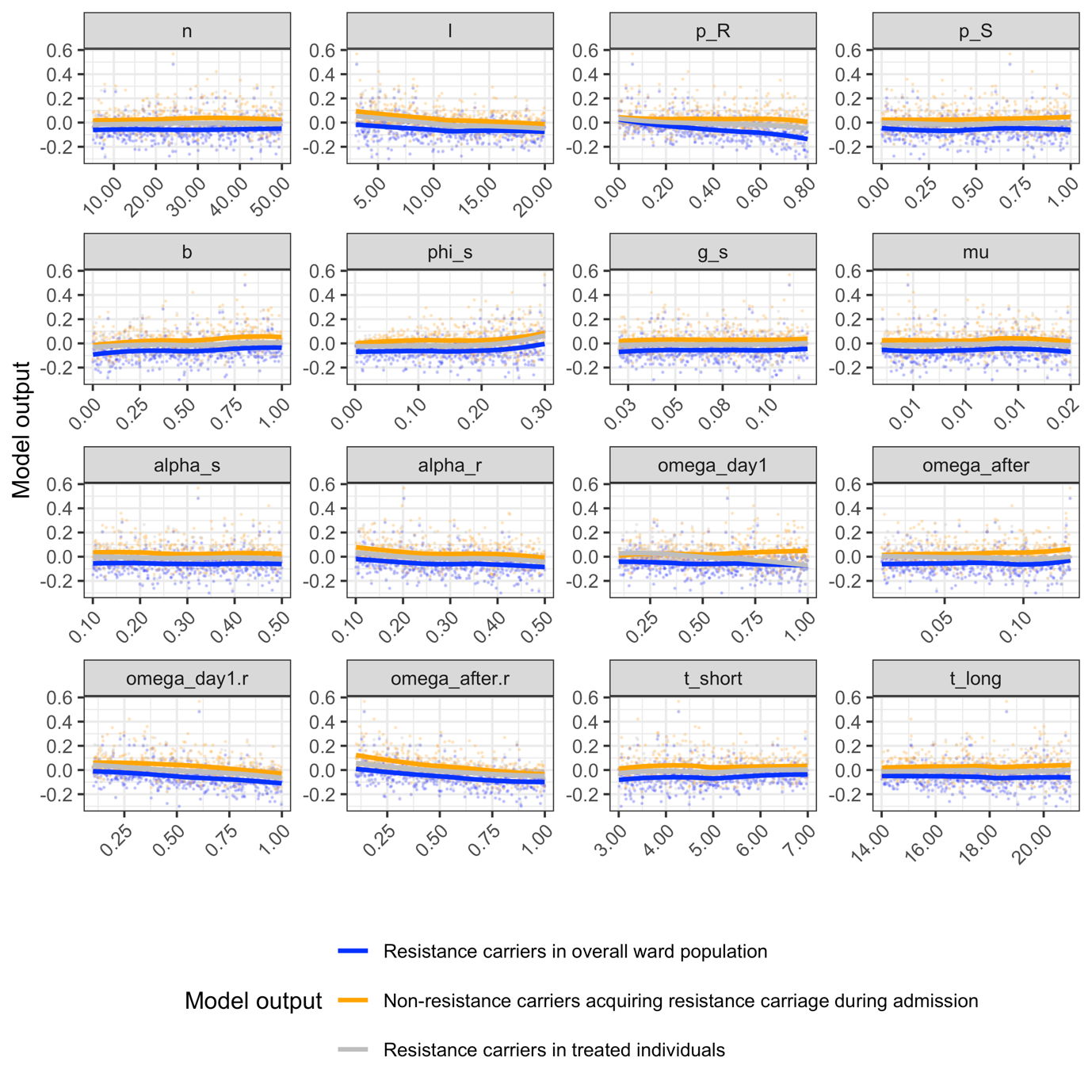


1. Co-colonisation model


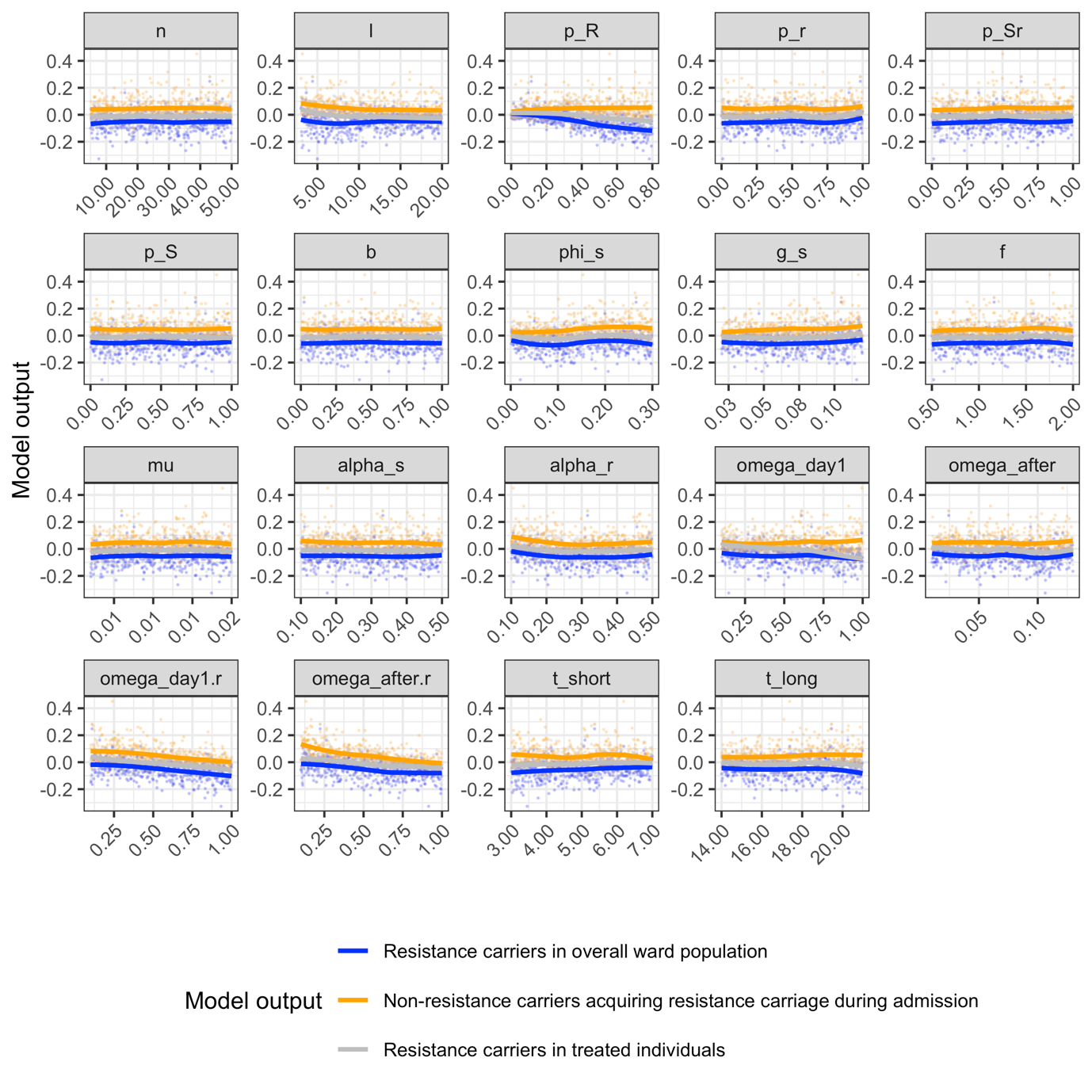


1. Within-host growth model


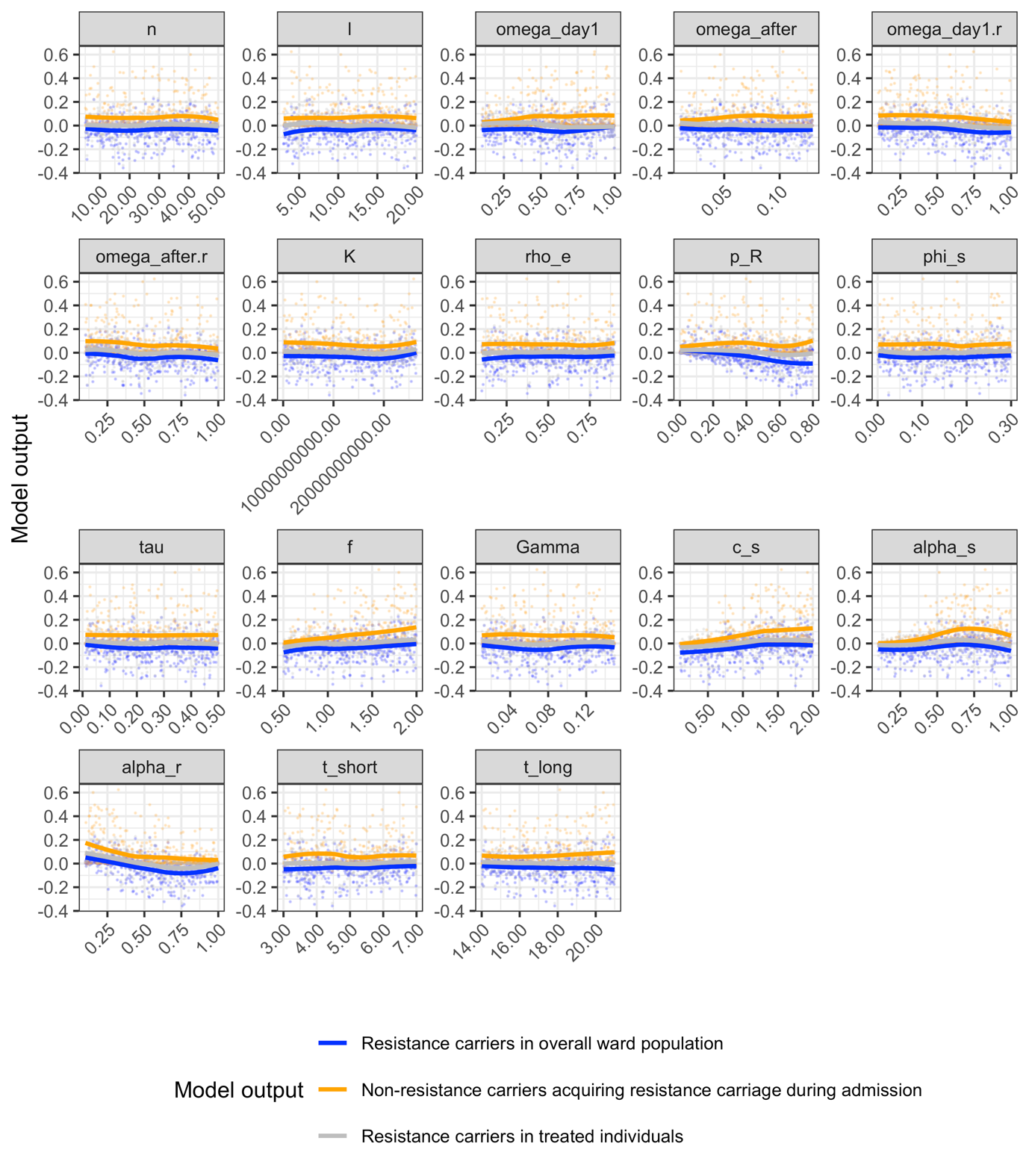


Fig C. Scatter plots of the parameters with the output from the three models. Each dot represents the model output, either in terms of proportion of resistance carriers (blue) or proportion of patients who were admitted as non-resistance carriers but gained resistance colonisation (orange). The lines are regression lines obtained from locally estimated scatterplot smoothing (local regression fitting) with span 0.75. Details of each parameter can be found in Table B in S1 Text. Similar graphs were produced using 10 other sets of 500 unique sets of parameters.

- Hoeffding's D measure and Spearman's rank correlation measures

Spearman's rank correlation indicated the strength and direction of a relationship between two variables and falls between 1 and -1. Spearman's correlation can be applied to a non-monotonic relationship to determine if there is a monotonic component to the association.

Hoeffding's D measure tests the independence of the data sets by calculating the distance between the product of the marginal distributions under the null hypothesis and the empirical bivariate distribution. Unlike the Pearson or Spearman measures, it detects non-linear relationships. Hoeffding’s D lies on the interval [-0.5, 1] if there are no tied ranks, with larger values indicating a stronger relationship between the variables.

Table B. Hoeffding's D measure and Spearman's rank correlation measure. The numbers in the brackets refer to the p-values calculated with 0.05 type 1 error.

|  |  | **Exclusive colonisation model** | | **Co-colonisation model** | | **Within-host growth model** | |
| --- | --- | --- | --- | --- | --- | --- | --- |
| **Parameter** | **Outcome** | **HD** | **SPM** | **HD** | **SPM** | **HD** | **SPM** |
| **n** | R carriers per day | 0 (0.14) | 0.08 (0.10) | 0 (0.70) | 0.02 (0.67) | 0 (0.29) | 0.03 (0.49) |
|  | New R acquisitions per admission | 0 (0.57) | 0.02 (0.70) | 0 (0.62) | 0.02 (0.62) | 0 (0.59) | 0.01 (0.80) |
| **l** | R carriers per day | 0.01 (<0.01) | 0.14 (0.01) | 0 (0.73) | 0.01 (0.91) | 0 (0.50) | -0.01 (0.77) |
|  | New R acquisitions per admission | 0.01 (<0.01) | -0.22 (<0.01) | 0 (0.09) | -0.10 (0.04) | 0 (0.14) | 0.07 (0.15) |
| **p_R** | R carriers per day | 0.25 (<0.01) | 0.76 (<0.01) | 0.46 (<0.01) | 0.91 (<0.01) | 0.55 (<0.01) | 0.93 (<0.01) |
|  | New R acquisitions per admission | 0 (0.29) | 0 (0.97) | 0 (0.05) | 0.10 (0.04) | 0 (0.23) | 0.01 (0.92) |
| **p_S** | R carriers per day | 0 (0.21) | -0.06 (0.24) | 0 (0.21) | -0.03 (0.54) | - | - |
|  | New R acquisitions per admission | 0 (0.09) | 0.09 (0.05) | 0 (0.48) | 0.02 (0.73) | - | - |
| **b** | R carriers per day | 0.01 (<0.01) | -0.18 (<0.01) | 0 (0.76) | -0.01 (0.83) | - | - |
|  | New R acquisitions per admission | 0.01 (<0.01) | 0.17 (<0.01) | 0 (0.44) | 0.02 (0.70) | - | - |
| **phi_s** | R carriers per day | 0.06 (<0.01) | 0.42 (<0.01) | 0 (0.55) | 0.03 (0.53) | 0 (0.55) | 0.01 (0.81) |
|  | New R acquisitions per admission | 0.03 (<0.01) | 0.12 (0.01) | 0.01 (<0.01) | 0.17 (<0.01) | 0 (0.08) | -0.05 (0.31) |
| **g_s** | R carriers per day | 0 (0.36) | -0.05 (0.34) | 0 (0.05) | 0.11 (0.03) | - | - |
|  | New R acquisitions per admission | 0 (0.46) | 0 (0.99) | 0.01 (<0.01) | 0.19 (<0.01) | - | - |
| **mu** | R carriers per day | 0 (0.03) | -0.09 (0.07) | 0 (0.11) | -0.07 (0.17) | - | - |
|  | New R acquisitions per admission | 0 (0.27) | 0.07 (0.14) | 0 (0.65) | 0.02 (0.67) | - | - |
| **alpha_s** | R carriers per day | 0 (0.34) | 0.01 (0.80) | 0 (0.47) | 0.03 (0.50) | 0 (0.10) | 0.08 (0.09) |
|  | New R acquisitions per admission | 0 (0.49) | -0.04 (0.47) | 0 (0.12) | -0.06 (0.20) | 0.06 (<0.01) | 0.36 (<0.01) |
| **alpha_r** | R carriers per day | 0 (0.03) | -0.11 (0.03) | 0 (0.05) | -0.11 (0.02) | 0.01 (<0.01) | -0.18 (<0.01) |
|  | New R acquisitions per admission | 0.01 (<0.01) | -0.17 (<0.01) | 0 (0.15) | -0.09 (0.07) | 0 (0.01) | -0.13 (0.01) |
| **omega_day1** | R carriers per day | 0 (0.17) | -0.09 (0.07) | 0 (0.08) | -0.08 (0.09) | 0 (0.22) | -0.04 (0.37) |
|  | New R acquisitions per admission | 0.01 (<0.01) | 0.11 (0.02) | 0.03 (<0.01) | 0.23 (<0.01) | 0.01 (<0.01) | 0.22 (<0.01) |
| **omega_after** | R carriers per day | 0 (0.14) | 0.08 (0.11) | 0 (0.24) | 0.03 (0.56) | 0 (0.47) | 0.01 (0.91) |
|  | New R acquisitions per admission | 0 (0.25) | -0.07 (0.14) | 0 (0.36) | -0.06 (0.24) | 0 (0.41) | -0.02 (0.66) |
| **omega_day1.r** | R carriers per day | 0 (0.02) | -0.13 (0.01) | 0.01 (<0.01) | -0.17 (<0.01) | 0 (0.02) | -0.10 (0.05) |
|  | New R acquisitions per admission | 0.22 (<0.01) | -0.75 (<0.01) | 0.25 (<0.01) | -0.77 (<0.01) | 0.03 (<0.01) | -0.34 (<0.01) |
| **omega_after.r** | R carriers per day | 0 (0.80) | -0.01 (0.88) | 0 (0.63) | 0 (0.94) | 0 (0.45) | -0.01 (0.86) |
|  | New R acquisitions per admission | 0 (0.57) | -0.01 (0.82) | 0 (0.09) | -0.07 (0.13) | 0 (0.50) | -0.03 (0.49) |
| **t_short** | R carriers per day | 0 (0.51) | -0.01 (0.82) | 0 (0.52) | -0.02 (0.66) | 0 (0.17) | -0.01 (0.86) |
|  | New R acquisitions per admission | 0 (0.47) | -0.04 (0.44) | 0 (0.16) | -0.03 (0.52) | 0 (0.12) | -0.02 (0.68) |
| **t_long** | R carriers per day | 0 (0.65) | -0.01 (0.78) | 0 (0.50) | 0.01 (0.91) | 0 (0.54) | -0.01 (0.92) |
|  | New R acquisitions per admission | 0 (0.05) | 0.08 (0.09) | 0 (0.05) | 0.08 (0.08) | 0 (0.14) | 0.10 (0.04) |
| **p_r** | R carriers per day | - | - | 0 (0.04) | 0.11 (0.03) | - | - |
|  | New R acquisitions per admission | - | - | 0 (0.01) | 0.12 (0.01) | - | - |
| **p_Sr** | R carriers per day | - | - | 0 (0.04) | -0.09 (0.06) | - | - |
|  | New R acquisitions per admission | - | - | 0 (0.17) | 0.06 (0.24) | - | - |
| **f** | R carriers per day | - | - | 0 (0.04) | 0.10 (0.05) | 0 (0.49) | 0.03 (0.51) |
|  | New R acquisitions per admission | - | - | 0 (0.05) | 0.10 (0.04) | 0.03 (<0.01) | 0.31 (<0.01) |
| **K** | R carriers per day | - | - | - | - | 0 (0.53) | -0.01 (0.84) |
|  | New R acquisitions per admission | - | - | - | - | 0 (0.13) | -0.08 (0.12) |
| **rho_e** | R carriers per day | - | - | - | - | 0 (0.41) | -0.06 (0.23) |
|  | New R acquisitions per admission | - | - | - | - | 0 (0.39) | -0.06 (0.26) |
| **Gamma** | R carriers per day | - | - | - | - | 0 (0.10) | -0.08 (0.10) |
|  | New R acquisitions per admission | - | - | - | - | 0 (0.18) | -0.08 (0.10) |
| **tau** | R carriers per day | - | - | - | - | 0 (0.45) | 0.04 (0.47) |
|  | New R acquisitions per admission | - | - | - | - | 0 (0.30) | 0.07 (0.16) |
| **c_s** | R carriers per day | - | - | - | - | 0 (0.53) | 0.03 (0.52) |
|  | New R acquisitions per admission | - | - | - | - | 0.07 (<0.01) | 0.44 (<0.01) |

HD: Hoeffding's D measure

SPM: Spearman's rank correlation

**References**

1. Lübbert C, Faucheux S, Becker-Rux D, Laudi S, Dürrbeck A, Busch T, et al. Rapid emergence of secondary resistance to gentamicin and colistin following selective digestive decontamination in patients with KPC-2-producing Klebsiella pneumoniae: A single-centre experience. Int J Antimicrob Agents. 2013;42: 565–570. doi:10.1016/j.ijantimicag.2013.08.008

2. Paul M, Daikos GL, Durante-Mangoni E, Yahav D, Carmeli Y, Benattar YD, et al. Colistin alone versus colistin plus meropenem for treatment of severe infections caused by carbapenem-resistant Gram-negative bacteria: an open-label, randomised controlled trial. Lancet Infect Dis. 2018;18: 391–400. doi:10.1016/S1473-3099(18)30099-9

3. Arcilla MS, van Hattem JM, Haverkate MR, Bootsma MCJ, van Genderen PJJ, Goorhuis A, et al. Import and spread of extended-spectrum β-lactamase-producing Enterobacteriaceae by international travellers (COMBAT study): a prospective, multicentre cohort study. Lancet Infect Dis. 2017;17: 78–85. doi:10.1016/S1473-3099(16)30319-X

4. Van Tongeren SP, Slaets JPJ, Harmsen HJM, Welling GW. Fecal microbiota composition and frailty. Appl Environ Microbiol. 2005;71: 6438–6442. doi:10.1128/AEM.71.10.6438-6442.2005

5. Niehus R, Van Kleef E, Yin M, Turlej-Rogacka​ A, Lammens C, Carmeli Y, et al. Quantifying antibiotic impact on within-host dynamics of extended-spectrum beta-lactamase resistance in hospitalized patients. 2015; 1–21. doi:10.1101/548453

6. Rinninella E, Raoul P, Cintoni M, Franceschi F, Miggiano GAD, Gasbarrini A, et al. What is the healthy gut microbiota composition? A changing ecosystem across age, environment, diet, and diseases. Microorganisms. 2019;7. doi:10.3390/microorganisms7010014

7. Palleja A, Mikkelsen KH, Forslund SK, Kashani A, Allin KH, Nielsen T, et al. Recovery of gut microbiota of healthy adults following antibiotic exposure. Nat Microbiol. 2018;3: 1255–1265. doi:10.1038/s41564-018-0257-9

8. Shaw LP, Bassam H, Barnes CP, Walker AS, Klein N, Balloux F. Modelling microbiome recovery after antibiotics using a stability landscape framework. ISME J. 2019;13: 1845–1856. doi:10.1038/s41396-019-0392-1

9. Zaura E, Brandt BW, de Mattos MJT, Buijs MJ, Caspers MPM, Rashid MU, et al. Same Exposure but two radically different responses to antibiotics: Resilience of the salivary microbiome versus long-term microbial shifts in feces. MBio. 2015;6. doi:10.1128/mBio.01693-15

10. Gibson B, Wilson DJ, Feil E, Eyre-Walker A. The distribution of bacterial doubling times in the wild. Proc R Soc B Biol Sci. 2018;285. doi:10.1098/rspb.2018.0789

11. Tängdén T, Cars O, Melhus Å, Löwdin E. Foreign travel is a major risk factor for colonization with Escherichia coli producing CTX-M-type extended-spectrum β-lactamases: A prospective study with Swedish volunteers. Antimicrob Agents Chemother. 2010;54: 3564–3568. doi:10.1128/AAC.00220-10

12. Hilty M, Betsch BY, Bögli-Stuber K, Heiniger N, Stadler M, Küffer M, et al. Transmission dynamics of extended-spectrum -lactamase-producing enterobacteriaceae in the tertiary care hospital and the household setting. Clin Infect Dis. 2012;55: 967–975. doi:10.1093/cid/cis581

13. Tran DM, Larsson M, Olson L, Hoang NTB, Le NK, Khu DTK, et al. High prevalence of colonisation with carbapenem-resistant Enterobacteriaceae among patients admitted to Vietnamese hospitals: Risk factors and burden of disease. J Infect. 2019;79: 115–122. doi:10.1016/j.jinf.2019.05.013

14. Bar-Yoseph H, Hussein K, Braun E, Paul M. Natural history and decolonization strategies for ESBL/carbapenem-resistant Enterobacteriaceae carriage: Systematic review and meta-analysis. Journal of Antimicrobial Chemotherapy. Oxford University Press; 2016. pp. 2729–2739. doi:10.1093/jac/dkw221

15. Kager L, Brismar B, Malmborg AS, Nord CE. Imipenem concentrations in colorectal surgery and impact on the colonic microflora. Antimicrob Agents Chemother. 1989;33: 204–208. doi:10.1128/AAC.33.2.204

16. Bächer K, Schaeffer M, Lode H, Nord CE, Borner K, Koeppe P. Multiple dose pharmacokinetics, safety, and effects on faecal microflora, of cefepime in healthy volunteers. J Antimicrob Chemother. 1992;30: 365–375. doi:10.1093/jac/30.3.365

17. Nord CE, Nord CE, Brismar B, Kasholm-Tengve B, Tunevall G. Effect of piperacillin/tazobactam treatment on human bowel microflora. J Antimicrob Chemother. 1993;31: 61–65. doi:10.1093/jac/31.suppl_A.61

18. McKay MD, Beckman RJ, Conover WJ. Comparison of three methods for selecting values of input variables in the analysis of output from a computer code. Technometrics. 1979;21: 239–245. doi:10.1080/00401706.1979.10489755

19. Saltelli A, Marivoet J. Non-parametric statistics in sensitivity analysis for model output: A comparison of selected techniques. Reliab Eng Syst Saf. 1990;28: 229–253. doi:10.1016/0951-8320(90)90065-U

20. Santos S de S, Takahashi DY, Nakata A, Fujita A. A comparative study of statistical methods used to identify dependencies between gene expression signals. Brief Bioinform. 2013;15: 906–918. doi:10.1093/bib/bbt051

21. Marino S, Hogue IB, Ray CJ, Kirschner DE. A methodology for performing global uncertainty and sensitivity analysis in systems biology. Journal of Theoretical Biology. J Theor Biol; 2008. pp. 178–196. doi:10.1016/j.jtbi.2008.04.011

22. Genest C, Plante J-F. On blest’s measure of rank correlation. Can J Stat. 2003;31: 35–52. doi:10.2307/3315902

23. Read M, Andrews PS, Timmis J, Kumar V. Techniques for grounding agent-based simulations in the real domain: a case study in experimental autoimmune encephalomyelitis. Math Comput Model Dyn Syst. 2012;18: 67–86. doi:10.1080/13873954.2011.601419

24. Vargha A, Delaney HD. A critique and improvement of the CL common language effect size statistics of McGraw and Wong. J Educ Behav Stat. 2000;25: 101–132. doi:10.3102/10769986025002101
