# Supplementary material for: Implications of reducing antibiotic treatment duration for antimicrobial resistance in hospital settings: A modelling study and meta-analysis
Source: PLoS Med. 2023 Jun 15;20(6):e1004013. doi: 10.1371/journal.pmed.1004013 (PMC10270346; doi:10.1371/journal.pmed.1004013)

**Supplementary material 2: Systematic review methodology**

**S2.1 Systematic search strategy**

A comprehensive, electronic search strategy was used to identify randomised controlled trials, published from 1 January 2000 up to 4 October 2022, and indexed in MEDLINE and EMBASE.[1] Searches were re-ran prior to the final analysis. Unpublished

studies were not sought.

- Review question

How do antibiotic treatments, when given in various durations, affect the colonisation status of the treated patients by antibiotic resistant bacteria?

- Inclusion criteria:

1. Participants: Patients from both hospital and community settings who received antibiotics for reasons including but not limited to the prevention or treatment of infections or inflammatory illnesses.
2. Intervention: Varying durations of antibiotic treatment. This refers to continuous duration of antibiotic course (as oppose to interrupted courses). The specific indirect interventions which guide antibiotic prescriptions or duration of treatment may include the use of inflammatory markers.
3. Comparison: Patients who received antibiotics of a different duration.
4. Outcome: Number of patients who were colonised with antibiotic resistant bacteria before and after antibiotic treatment. The sites of colonisation may include, but not limited to, the digestive tract, respiratory tract, urinary tract.

**Table A: Search terms used in the systematic review**.

| Database | Search terms |
| --- | --- |
| MEDLINE | ((antibiotic) AND (infection)) AND (weeks[Title]) Filters: Randomized Controlled Trial, Humans, from 2000 – 2022  Details: (((((((("anti bacterial agents"[Pharmacological Action] OR "anti-bacterial agents"[MeSH Terms]) OR ("anti bacterial"[All Fields] AND "agents"[All Fields])) OR "anti bacterial agents"[All Fields]) OR "antibiotic"[All Fields]) OR "antibiotics"[All Fields]) OR "antibiotic s"[All Fields]) OR "antibiotical"[All Fields]) AND ((((((((((((((((((((("infect"[All Fields] OR "infectability"[All Fields]) OR "infectable"[All Fields]) OR "infectant"[All Fields]) OR "infectants"[All Fields]) OR "infected"[All Fields]) OR "infecteds"[All Fields]) OR "infectibility"[All Fields]) OR "infectible"[All Fields]) OR "infecting"[All Fields]) OR "infection s"[All Fields]) OR "infections"[MeSH Terms]) OR "infections"[All Fields]) OR "infection"[All Fields]) OR "infective"[All Fields]) OR "infectiveness"[All Fields]) OR "infectives"[All Fields]) OR "infectivities"[All Fields]) OR "infects"[All Fields]) OR "pathogenicity"[MeSH Subheading]) OR "pathogenicity"[All Fields]) OR "infectivity"[All Fields])) AND "weeks"[Title] |
|  | ((antibiotic) AND (infection)) AND (days[Title]) Filters: Randomized Controlled Trial, Humans, from 2000 – 2022  Details: (((((((("anti bacterial agents"[Pharmacological Action] OR "anti-bacterial agents"[MeSH Terms]) OR ("anti bacterial"[All Fields] AND "agents"[All Fields])) OR "anti bacterial agents"[All Fields]) OR "antibiotic"[All Fields]) OR "antibiotics"[All Fields]) OR "antibiotic s"[All Fields]) OR "antibiotical"[All Fields]) AND ((((((((((((((((((((("infect"[All Fields] OR "infectability"[All Fields]) OR "infectable"[All Fields]) OR "infectant"[All Fields]) OR "infectants"[All Fields]) OR "infected"[All Fields]) OR "infecteds"[All Fields]) OR "infectibility"[All Fields]) OR "infectible"[All Fields]) OR "infecting"[All Fields]) OR "infection s"[All Fields]) OR "infections"[MeSH Terms]) OR "infections"[All Fields]) OR "infection"[All Fields]) OR "infective"[All Fields]) OR "infectiveness"[All Fields]) OR "infectives"[All Fields]) OR "infectivities"[All Fields]) OR "infects"[All Fields]) OR "pathogenicity"[MeSH Subheading]) OR "pathogenicity"[All Fields]) OR "infectivity"[All Fields])) AND "days"[Title] |
|  | ((antibiotic) AND (infection)) AND (duration) Filters: Randomized Controlled Trial, Humans, from 2000 – 2022  Details: (((((((("anti bacterial agents"[Pharmacological Action] OR "anti-bacterial agents"[MeSH Terms]) OR ("anti bacterial"[All Fields] AND "agents"[All Fields])) OR "anti bacterial agents"[All Fields]) OR "antibiotic"[All Fields]) OR "antibiotics"[All Fields]) OR "antibiotic s"[All Fields]) OR "antibiotical"[All Fields]) AND ((((((((((((((((((((("infect"[All Fields] OR "infectability"[All Fields]) OR "infectable"[All Fields]) OR "infectant"[All Fields]) OR "infectants"[All Fields]) OR "infected"[All Fields]) OR "infecteds"[All Fields]) OR "infectibility"[All Fields]) OR "infectible"[All Fields]) OR "infecting"[All Fields]) OR "infection s"[All Fields]) OR "infections"[MeSH Terms]) OR "infections"[All Fields]) OR "infection"[All Fields]) OR "infective"[All Fields]) OR "infectiveness"[All Fields]) OR "infectives"[All Fields]) OR "infectivities"[All Fields]) OR "infects"[All Fields]) OR "pathogenicity"[MeSH Subheading]) OR "pathogenicity"[All Fields]) OR "infectivity"[All Fields])) AND ("duration"[All Fields] OR "durations"[All Fields]) |
| EMBASE | antibiotic AND infection AND week*:ti AND [randomized controlled trial]/lim AND [2000-2022]/py AND 'human'/de |
|  | antibiotic AND infection AND day*:ti AND [randomized controlled trial]/lim AND [2000-2022]/py AND 'human'/de |
|  | antibiotic AND infection AND duration AND [randomized controlled trial]/lim AND [2000-2022]/py AND 'human'/de |

- Data extraction

Bibliographies of primary studies and review articles meeting the inclusion criteria were searched manually to identify further eligible studies. Data on objective and outcomes were independently extracted by two review authors.

**S2.3 PRISMA diagram**

**
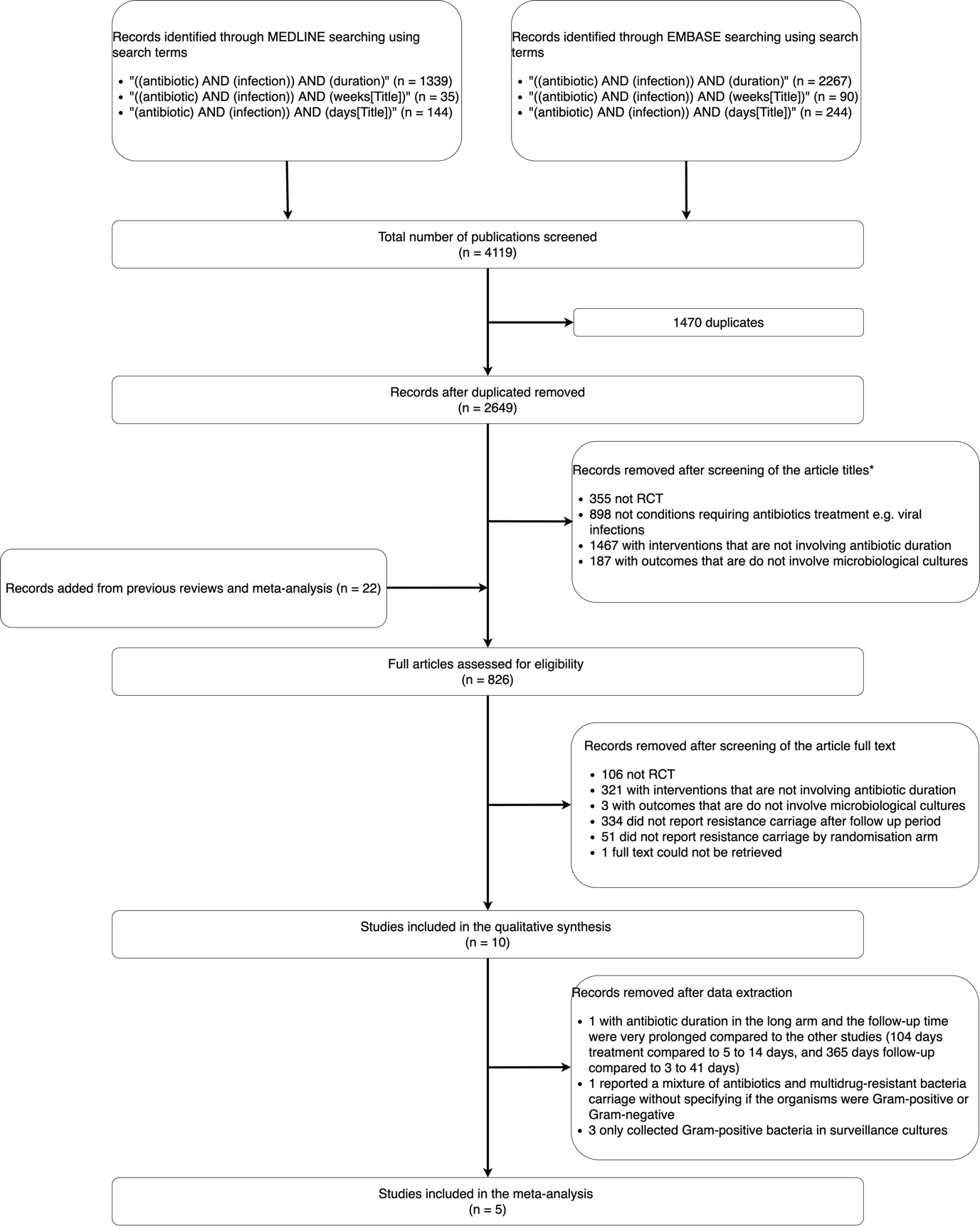
**

**Fig A. PRISMA diagram**

*Reasons for exclusion may overlap.

**S2.4 Meta-regression models**

The meta-regression analysis was performed using a Bayesian regression model to estimate the change in the daily risk of acquiring resistant bacteria colonisation per day of antibiotic consumption. A linear relationship was assumed between the log of the daily acquisition risk and days of antibiotic intake.

Let *p_ij_* be the daily probability for an individual to acquire colonisation by a resistant bacteria in arm *j* of study *i*.

The number of patients colonised with resistant bacteria is represented by *Y_ij_*, in arm *j* of study *i*. *Y_ij_* was modelled with a binomial distribution:

*Y_ij_* ∼ Bin(*n_ij_, p_ij_*)

where *n_i,j_* is the total number of participants in arm *j* of trial *i,* and *p_ij_* is the probability of colonisation over the follow-up period.

We compared three models for *p_ij_*.

**Model 1**

logit(*p_ij_*) = *α_i_* + *β_i_t_ij_ + c_i_f_ij_ + d_i_w_ij_,*

where *t_ij_* is the duration of antibiotic used in arm *j* of trial *i* and *p_ij_* is the probability of colonisation over a follow-up period of *f_i_* days. The setting where the trial was conducted, i.e., inpatient or outpatient setting, was indicated by a binary variable, *w_ij_*.

**Model 2**

logit(*p_ij_*) = *α_i_* + *β_i_t_ij_ + c_i_f_ij_,*

In models 1 and 2, intercepts, *α_i_*, and slopes, *β_i,_ c_i_* and *d_i_*, were allowed to vary between trials and assumed to be normally distributed.

**Model 3**

logit(*p_ij_*) = *α_ij_* + *β_ij_t_ij_ + c_ij_f_ij_ + d_ij_w_ij_,*

In model 3, intercepts, *α_ij_*, and slopes, *β_ij_* and *c_ij_* were allowed to vary between arms of each trial and assumed to be independent and normally distributed.

We implemented the above meta-regression model in JAGS using the R2jags package,[2] and performed all analysis in R version 3.6.2[3].

### S2.5 Model assessment of the meta-analysis models

Prior distributions were selected to be weakly informative normal distributions. We assessed the models using measures of Markov chain convergence including effective sample sizes and *R*ˆ which indicate if the chains had run for long enough and had mixed well.

Plots of iterations vs. sampled values for model parameters in the MCMC chains. The three different chains are plotted using different colours.

In all the models, the *R*ˆ values were about 1 and the minimum effective sample size was 1000 across all parameters. The chains’ mixing from the chosen model is shown below.


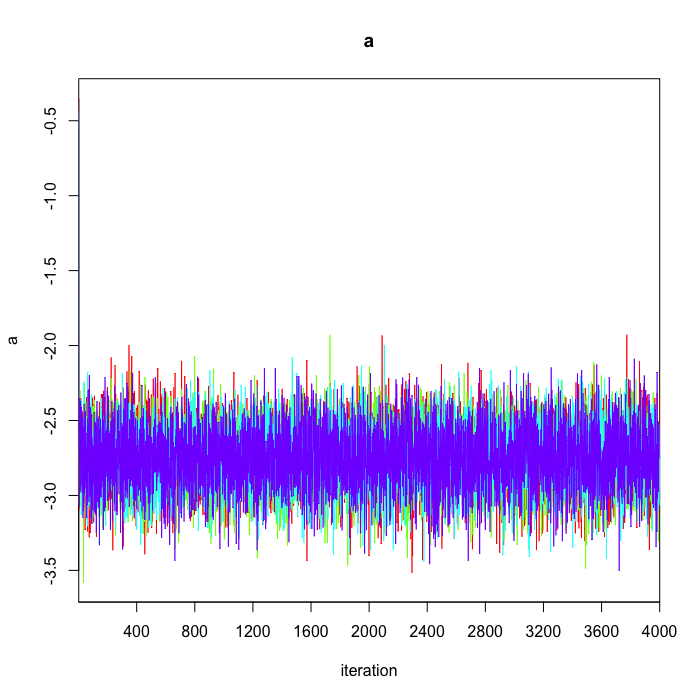

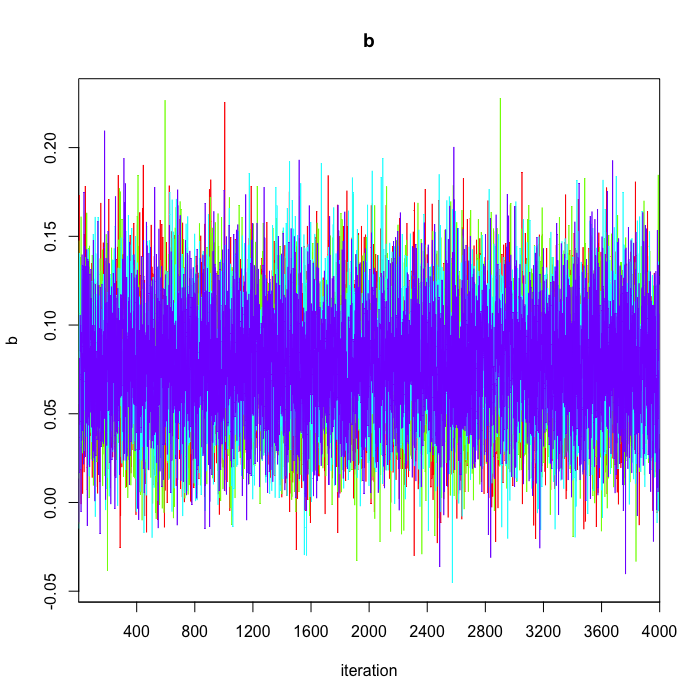

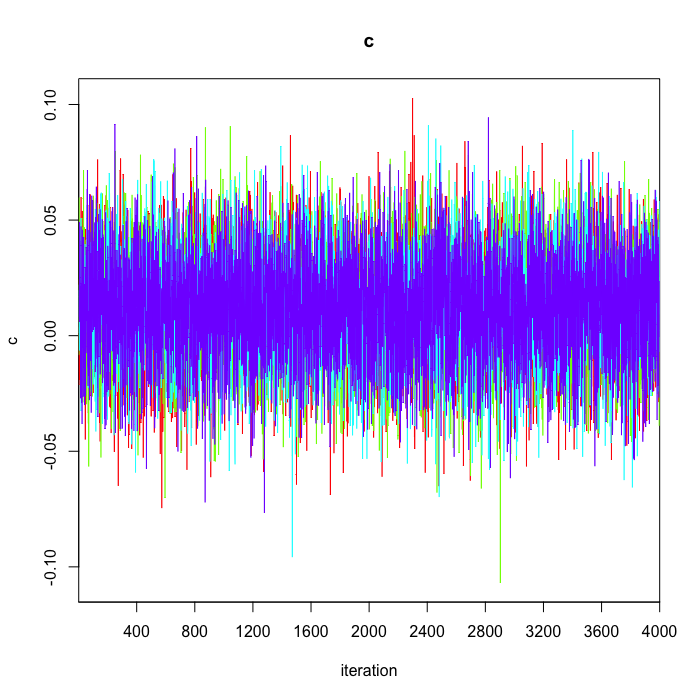


**S2.6 Model comparisons**

The three models were compared with the best fit to data by the Watanabe–Akaike information criterion (WAIC). The chosen model was the one which has an intercept (*α*), representing the baseline colonisation risk, and slopes (*b* and *c*) which represent colonisation risk associated with one additional day of antibiotic treatment and follow-up period respectively. In this model, both the intercepts (*α)* and slopes (*b* and *c*) varied by arms in each trial (no random effect given to the trials).

Table B. Comparison of widely applicable information criterion between the three models.

| Transmission model | Parameters | Priors | WAIC † |
| --- | --- | --- | --- |
| Trials as random effect | *α* (intercept)  *β* (effect of duration on resistance carriage)  *c* (effect of follow-up period on resistance carriage)  *d* (effect of healthcare setting on resistance carriage) | *normal*(0*,3)* | -0.0374 |
| No random effect | *α* (intercept)  *β* (effect of duration on resistance carriage)  *c* (effect of follow-up period on resistance carriage)  *d* (effect of healthcare setting on resistance carriage) | *normal*(0*,3)* | -0.1429 |
| No random effect, no effect of healthcare setting on resistance carriage | *α* (intercept)  *β* (effect of duration on resistance carriage)  *c* (effect of follow-up period on resistance carriage) | *normal*(0*,3)* | -0.1435 |

† Widely applicable information criterion (WAIC)

**S2.7 Sensitivity analysis**

Table C. Sensitivity analysis were performed with a 30-day cut-off for surveillance cultures and different priors.

|  | **Odds ratio for being colonised with resistant bacteria per additional day of antibiotic treatment (80% credible intervals)** |
| --- | --- |
| **Main analysis** | 1.08(1.04 to 1.12) |
| **30-day cut-off for surveillance cultures instead of 60-day** | 1.08(1.04 to 1.13) |
| **Using priors *normal*(0*,7)* instead of *normal*(0*,3)*** | 1.08(1.04 to 1.12) |

**Table D. Quality assessment of the randomised controlled trials included in the meta-analysis.**

| Reference | Bias arising from the randomisation process | Bias due to deviation from intended interventions | Bias due to missing outcome data | Bias in measurement of the outcome | Bias in selection of the reported result |
| --- | --- | --- | --- | --- | --- |
| Lutsar, 2020[4] | Some concerns (Median birth weight in standard-of-care group is lower than that of the meropenem group; other characteristics look similar) | Low risk of bias | Low risk of bias | High risk (The composite primary endpoint potentially subjective) | Some concerns (  Composite primary outcome) |
| Hoberman, 2016[5] | Low risk of bias | Low risk of bias | High risk (19 out of 257 those in 10 days treatment lost to follow up or consent withdrew vs 39 out of 258 in 5 days) | Low risk of bias | Low risk of bias |
| Ceran, 2010[6] | Low risk of bias | Low risk of bias | High risk (Urine sediments could not be obtained in 15 patients, 15 patients lost to follow-up on day 7, the urine sample was not adequate in 9 patients at first follow-up visit, and 12 patients did not complete the second-month visit; thus, 118 patients were excluded. The remaining 142 patients completed the study.) | Low risk of bias | Some concerns (  Composite primary outcome) |
| Merode, 2005[7] | Low risk of bias | Low risk of bias | High risk (94 out of 160 in 3-day arm had no urine sample and no questionnaire; 101 out of 164 in 5-day arm had no urine sample; but reasons unclear) | High risk (Primary outcome collected via questionnaire which is subjected to recall bias) | Some concerns (  Composite primary outcome) |
| Dow, 2004[8] | Low risk of bias | Low risk of bias | Low risk of bias | Low risk of bias | Low risk of bias |

**References**

1. Bramer WM, Rethlefsen ML, Kleijnen J, Franco OH. Optimal database combinations for literature searches in systematic reviews: A prospective exploratory study. Syst Rev. 2017;6: 1–12. doi:10.1186/s13643-017-0644-y

2. Hornik K, Leisch F, Zeileis A, Plummer M. JAGS: A Program for Analysis of Bayesian Graphical Models Using Gibbs Sampling. Available: http://www.ci.tuwien.ac.at/Conferences/DSC-2003/

3. R Core Team. A language and environment for statistical computing. Vienna; Austria: R Foundation for Statistical Computing; 2017.

4. Lutsar I, Chazallon C, Trafojer U, De Cabre VM, Auriti C, Bertaina C, et al. Meropenem vs standard of care for treatment of neonatal late onset sepsis (NeoMero1): A randomised controlled trial. PLoS One. 2020;15: e0229380. doi:10.1371/journal.pone.0229380

5. Hoberman A, Paradise JL, Rockette HE, Kearney DH, Bhatnagar S, Shope TR, et al. Shortened Antimicrobial Treatment for Acute Otitis Media in Young Children. N Engl J Med. 2016;375: 2446–2456. doi:10.1056/nejmoa1606043

6. Ceran N, Mert D, Kocdogan FY, Erdem I, Adalati R, Ozyurek S, et al. A randomized comparative study of single-dose fosfomycin and 5-day ciprofloxacin in female patients with uncomplicated lower urinary tract infections. J Infect Chemother. 2010;16: 424–430. doi:10.1007/s10156-010-0079-z

7. van Merode T, Nys S, Raets I, Stobberingh EE. Acute uncomplicated lower urinary tract infections in general practice: Clinical and microbiological cute rates after three- versus five-day treatment with trimethoprim. Eur J Gen Pract. 2005;11: 55–58. doi:10.3109/13814780509178238

8. Dow G, Rao P, Harding G, Brunka J, Kennedy J, Alfa M, et al. A prospective, randomized trial of 3 or 14 days of ciprofloxacin treatment for acute urinary tract infection in patients with spinal cord injury. Clin Infect Dis. 2004;39: 658–664. doi:10.1086/423000

**S2.9 Funnel plot for assessment of publication bias**

In this funnel plot, the absolute difference between the proportion of resistance carriers in those who received long versus short antibiotic treatment duration (x-axis) is plotted against the square root sample size (y-axis). However, it should be noted that in these 5 trials, antibiotic treatment days in the short and long antibiotic treatment arms differ. Hence, while this plot gives an idea of antibiotic treatment effect on resistance carriage in these 5 publications, the crude absolute difference in proportion of resistance carriers cannot be compared directly without accounting for the antibiotic treatment days. This underpins the rationale behind our meta-analysis, which used antibiotic treatment days in each randomisation arm as an independent variable.


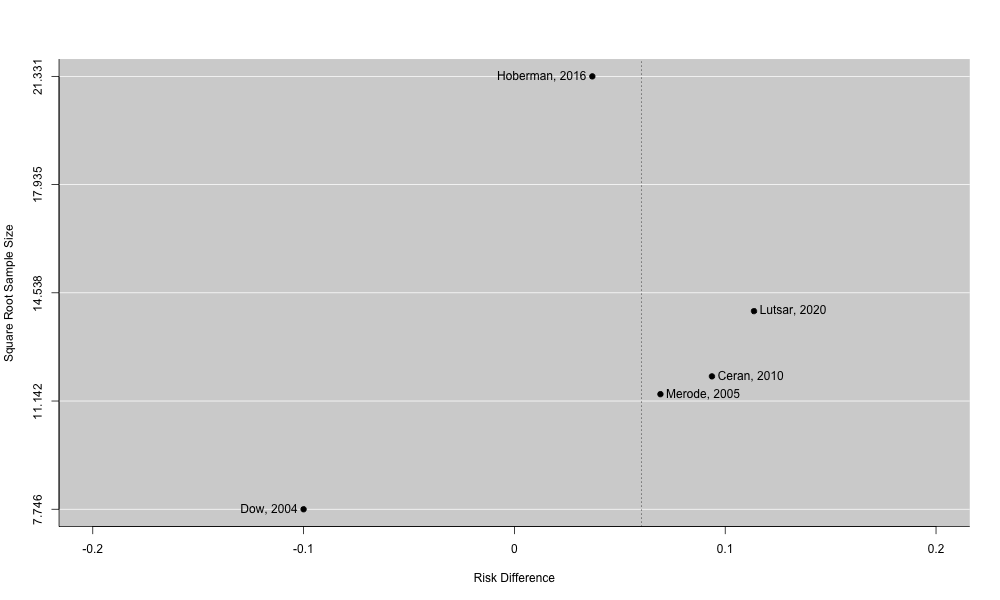

Supplement: S2 Text — Table A. Search terms used in the systematic review. Table B. Model comparisons. Table C. Sensitivity analysis. Table D. Quality assessment of the randomised controlled trials included in the meta-analysis. (DOCX) [file pmed.1004013.s002.docx]
