# Supplementary material for: Implications of reducing antibiotic treatment duration for antimicrobial resistance in hospital settings: A modelling study and meta-analysis
Source: PLoS Med. 2023 Jun 15;20(6):e1004013. doi: 10.1371/journal.pmed.1004013 (PMC10270346; doi:10.1371/journal.pmed.1004013)
Supplement: Attachment — Submitted filename: abm_abxdur_PlosMed_reply.docx [file pmed.1004013.s004.docx]

Professor Raffaella Bosurgi

*Editor-in-Chief*

*PLoS Medicine*

25 July 2022

Dear Professor Bosurgi,

**Re: Manuscript submission: “The effect of reducing antibiotic treatment duration on antimicrobial resistance in the healthcare setting: a modelling study and meta-analysis” for PLoS Medicine**

Thank you very much for the reviewers’ and the editorial board’s insightful comments, and the opportunity to revise our manuscript. Please find the accompanying revised manuscript for your review and consideration. Our responses to the comments are as follows.

**Comments from Editors**

- **General**

**Thank you for including the PRISMA diagram. Please also provide the completed PRISMA checklist. When completing the checklist, please use section and paragraph numbers, rather than page numbers.**

Please see the attached PRISMA checklist.

**We ask you to adapt the title to better match journal style, and suggest: "Implications of reducing antibiotic treatment duration for antimicrobial resistance in hospital settings: A modelling study and meta-analysis".**

We have changed the title according to the above suggestion.

- **Abstract**

**Please structure your abstract using the PLOS Medicine headings (Background, Methods and Findings, Conclusions).**

**Abstract Background: The final sentence should clearly state the study question.**

**Please combine the Methods and Findings sections into one section, “Methods and findings”.**

We have amended the abstract according to the above suggestions.

**Abstract Methods and Findings:**

**Please ensure that all numbers presented in the abstract are present and identical to numbers presented in the main manuscript text (i.e 187 studies Vs 7 included in analyses).**

We indicated in the abstract that “We identified 187 antibiotic duration randomised trials. Five reported resistant Gram-negative bacteria carriage as an outcome and were included in the meta-analysis.” which is consistent with the main text.

**Please include the study design and main outcome measures.**

For study design, we included in the abstract: “We constructed three stochastic mechanistic models that considered both between- and within-host dynamics of susceptible and resistant Gram-negative bacteria, to identify circumstances under which shortening antibiotic duration would lead to reduced resistance carriage. In addition, we performed a meta-analysis of randomised trials of antibiotic treatment duration.”

For outcome measures, we added: “In addition, we performed a meta-analysis of antibiotic treatment duration trials which monitored resistant Gram-negative bacteria carriage as an outcome.”

**Please include the important dependent variables that are adjusted for in the analyses.**

We added: “The meta-analysis was performed with a logistic regression using antibiotic duration administered to the patients in each arm and elapsed time from antibiotic administration to surveillance culture as independent variables.”

**In the last sentence of the Abstract Methods and Findings section, please describe the main limitation(s) of the study's methodology.**

We added: “Interpretation of these estimates is limited by the low number of antibiotic duration trials which monitored resistance carriage as an outcome, contributing to a large credible interval.”

**Please report your abstract according to PRISMA for abstracts, following the PLOS Medicine abstract structure (Background, Methods and Findings, Conclusions)**

**Please provide the dates of search, data sources, number of studies included, types of study designs included, eligibility criteria, and synthesis/appraisal methods**

We have added further details to the abstract to meet the PRISMA abstract requirements, while following the PLOS Medicine abstract structure.

**Abstract Conclusions:**

**Please address the study implications without overreaching what can be concluded from the data; the phrase "In this study, we observed ..." may be useful.**

**Please interpret the study based on the results presented in the abstract, emphasizing what is new without overstating your conclusions.**

We added the above phrase as suggested: “In this study, we found both theoretical and empirical evidence that reducing antibiotic treatment duration can reduce resistance carriage, though the mechanistic models also highlighted circumstances under which reducing treatment duration can, perversely, increase resistance.”

**After the abstract, please add a new and accessible 'Author summary' section in non-identical prose. You may find it helpful to consult one or two recent research papers in PLOS Medicine to get a sense of the preferred style.**

We have added an “Author summary” section as follows:

*Why was this study done?*

·	Shortening antibiotic treatment duration is a commonly adopted antibiotic stewardship strategy, with the expectation that it will reduce antimicrobial resistance in treated individuals and in the overall population.

·	Antibiotic selective pressure acts predominantly on ‘bystander’ colonising bacteria for resistance, and this depends on the agent’s spectrum of coverage, pharmacokinetic and pharmacodynamic properties, and bioavailability where the bacteria are located.

·	Empirical evidence and mechanistic understanding for how shortening antibiotic duration affects the emergence and spread of antimicrobial resistance is lacking.

·	Understanding the key factors driving the effect antibiotic duration on resistance carriage will inform future research study designs, antimicrobial stewardship interventions and resource allocation in the multimodal control strategies.

*What did the researchers do and find?*

·	We modelled within- and between-host dynamics of colonising ‘bystander’ susceptible and resistant bacteria in response to systemic antibiotic treatment, and compared the model findings with a systematic review and meta-analysis.

·	The models showed that shortening duration is most effective at reducing resistance carriage in high transmission settings.

·	For treated individuals, shortening duration is most effective when resistant bacteria grow rapidly under antibiotic selection pressure and decline rapidly when stopping treatment.

·	Shortening antibiotic treatment may increase resistance carriage when antibiotics administered can effectively eliminate the colonising bacteria with a particular resistance phenotype.

·	The meta-analysis found one additional antibiotic treatment day is associated with a 7% absolute increase in risk of resistance carriage when antibiotics administered were not effective against the resistance phenotype in the colonising bacteria.

*What do these findings mean?*

·	Shortening antibiotic duration may increase or decrease resistant bacteria colonisation depending on how effectively the resistant bacteria can be eliminated by antibiotics.

·	The modest effect of shortening treatment duration on resistant bacteria colonisation can be partially explained by typically short hospitalisation stays and a slow rate of decolonisation of resistant bacteria in patients previously exposed to antibiotics.

·	We found that shortening antibiotic treatment and reducing transmission of resistant bacteria through infection prevention and control share an important complementary interaction relationship.

·	The main limitations were that some parameter values used in the model were not available in the literature or were taken from animal or in vitro experiments, and for the meta-analysis, there were few antibiotic duration trials which monitored resistance carriage as an outcome.

- **Main manuscript**

**Please evaluate study quality and risk of bias.**

**Please evaluate evidence of publication bias.**

We have performed quality assessment of the studies using the Cochrane risk-of-bias tool for randomised trials (RoB 2 tool). Results are discussed in Results section paragraph 5: “Three out of five studies were deemed to be at risk of bias due to the significant proportion of participants who were lost to follow up and did not provide surveillance samples. However all studies were assessed to be at low risk of bias due to deviation from intended interventions.”

A summary table is provided in Supplementary material 2 S2.9.

**Please add the following statement, or similar, to the Methods: "This study is reported as per the Preferred Reporting Items for Systematic Reviews and Meta-Analyses (PRISMA) guideline (S1 Checklist)."**

We have added the above sentence in the Methods section.

**Please clarify the discrepancy between the in the number of studies reported to be included in the analysis (7) and the number of studies referenced in the supp data (9) and provide an updated list as necessary**

We have attached an updated PRISMA diagram with 7 shortlisted studies.

**Please remove COI/funding source/data sharing info from the end of the manuscript and instead include in the manuscript submission form. Please also remove the 'Role of the funding source' statement from the end of the Methods section.**

We have removed the above sections.

**At line 523, "... systematic review"?**

Thank you for spotting this. We have corrected this spelling error.

**Throughout the text, please style reference call-outs as follows: "... of resistance [5,6]." (noting the absence of spaces within the square brackets).**

**In the reference list, please convert all italics to plain text.**

**Where appropriate, please list 6 author names rather than 3, followed by "et al.".**

We have adjusted the references according to the above requirements.

- **Figures**

**Please provide titles and legends for all figures (including those in Supporting Information files).**

We have provided titles and legends for all figures.

**Please consider avoiding the use of red and green in order to make your figure more accessible to those with colour blindness**

We have adjusted all colours to avoid red and green.

**Comments from reviewer 1:**

**This interesting manuscript describes a modelling study that examines the impact of duration of antibiotic treatment on antibiotic resistance in Gram negative bacteria using three different agent-based models.**

**Strengths:**

**-	The manuscript is well written (which is not an easy task given the complexity of the topic) with very thorough methodology**

**-	Understanding the impact of duration of antibiotic therapy on antibiotic resistance is key to better inform antibiotic stewardship decisions**

**-	The manuscript nicely highlights how complex the relationship between treatment duration and antibiotic resistance is and where the knowledge gaps are**

**Weaknesses:**

**-	As with any model of this complexity a lot depends on the underlying assumptions which given the gaps in the literature remain speculative in many instances. The sensitivity analyses performed and the use of different model which give similar results are reassuring.**

**-	At times this manuscript is difficult to flow which is probably mainly a consequence of the complexity of the subject. At times simple figures may help understanding.**

We thank the reviewer for these positive comments! We acknowledge that the subject matter is complex. Figures 1 and 2 are simple illustrations which respectively presents an overview of the model structures for individual patients, and how the models are executed in the simulations, step-by-step.

- **Title**

**Minor comment: "The effect of reducing antibiotic treatment duration on antimicrobial resistance in the**

**healthcare setting" Hospital setting rather than healthcare setting is probably preferable?**

We have changed the title according to the editors’ comments.

- **Abstract**

**Major comment: While I understand that space is limited, I feel that the abstract could give more detail regarding the models and underlying assumptions. The way it is currently written it is difficult to even get a vague idea about what was done.**

Thank you for the feedback. We have added further details to the abstract according to PRISMA standards.

**Minor comment: The hypothetical setting should be described in the abstract.**

We have included this in the abstract: “We constructed three theoretical stochastic mechanistic models that considered both between- and within-host dynamics of susceptible and resistant Gram-negative bacteria, to identify circumstances under which shortening antibiotic duration would lead to reduced resistance carriage.”

**Minor comment: The term "resistance carriage" should be better defined since it is the main outcome of interest**

**Minor comment: "7% increase in risk of resistance carriage" Absolute or relative?**

Thank you for raising this. We have added “absolute” to the “7% increase in risk of resistance carriage” in the abstract and main text.

- **Introduction**

**Major comment: I think it would be good to add a paragraph in the introduction about the potential impact on transmission. The association of antibiotic treatment duration and length of stay (and thus probability of transmission / acquisition) would also be worth mentioning.**

Thank you for raising this comment. We have added the following according to the reviewer’s suggestions: “Emergence of resistance in colonising bacteria may be due to selection of resistant phenotypes that are present at the start of treatment but at low abundance or acquired by transmission during treatment; in a particular species as a result of horizontal gene transfer; or as a result of de-novo mutations. The effect of treatment duration on development of resistance will depend on the parameters governing these processes and their relative importance.”

**Major comment: It would also be important to mention that many of the resistant pathogens we care most about (e.g. carbapenemase and extended spectrum beta-lactamase producing Enterobacterales; carbapenem-resistant Acinetobacter) are not selected de novo, but rather acquired through transmission (Unlike fluoroquinolone resistance in Enterobacterales, one will not "create" a carbapenemase producing E. coli even when giving carbapenems for months). I understand that this intended by the "bystander" terminology but I think it would be good to give concrete examples.**

We thank the reviewer for this comment. We have added: “Instead we focused on pathogens in which colonisation with resistant phenotypes is primarily acquired through transmission and horizontal gene transfer. These are highly clinically relevant Gram-negative bacilli such as carbapenemase and extended spectrum beta-lactamase producing Enterobacterales and carbapenem-resistant *Acinetobacter* spp.” in the discussion section.

**Minor comment: "Examples of pathogens which may become resistant with inappropriate treatment include Mycobacterium tuberculosis and human immunodeficiency virus." Rather than using HIV as an example I would mention fluoroquinolone resistance in Enterobacterales.**

We have included “fluoroquinolone-resistant Enterobacterales” as an example: “Examples of pathogens which may become resistant during treatment include *Mycobacterium tuberculosis,* fluoroquinolone-resistant Enterobacterales and human immunodeficiency virus, especially with inappropriate dosing and duration”.

- **Methods and materials**

**Major comment: It would be good to describe the setup of the modelled hospital in more detail (I assume transfer between units was not simulated?)**

Thank you for this suggestion. We have clarified that: “In all models, we simulated individual patients in a single hospital ward environment.”

**Minor comment: "Within the same ward, antibiotics were prescribed with the same mean duration on or during admission." This is obviously quite an oversimplification and should be mentioned as a limitation. Why were the values not sampled from a distribution?**

We would like to clarify that antibiotics were prescribed with the same mean duration. The values were sampled from a normal distribution. We have added: “Exact duration for each antibiotic course was drawn from a normal distribution.”

**Minor comment "Real-world parameter values were obtained from the literature and used in these explorations (Supplementary material 1 Table 1.2)." These parameters are so essential for the interpretation of the model that they should be part of the main text. Also, some parameters have no references and it should be explained how they were derived.**

Thank you for this comment. We have added a subscript to the parameter value table (Supplementary material 1 Table 1.2) which explains: “References are provided for parameters values which could be found in the literature. For those parameter values not found in the literature, we explored the largest reasonable range of parameter values.” In addition, we have also added “For those parameter values not found in the literature, we explored the largest reasonable range of parameter values.” in the methods section. Example parameters which we did not provide references for include number of beds in the ward (5-50 beds), maximum length of stay for each patient in the ward (3-20 days), and probability of carrying resistant bacteria (1-80%). These ranges were purposefully kept large to inform in which particular setting shortening antibiotic duration was the most effective at reducing resistance carriage.

We did not include this table in the main text because there are already 8 figures and 1 table in the main text. We felt that the figures are more essential as they help to explain the model mechanisms and improve readability of the manuscript.

**Minor comment: Was the systematic review registered?**

We attempted to register the systematic review on PROSPERO. However due to the COVID-19 pandemic, COVID-19 relevant reviews were prioritised and the approval was delayed. We decided not to wait for this approval before commencing on the review.

- **Results:**

**Minor comment: "Key parameters in the relationship between antibiotic duration and resistance carriage" This section is quite difficult to understand. I acknowledge that is difficult to describe these complex findings simply, maybe some illustrations could help?**

Thank you for raising this point. We acknowledge that the models are complicated. We list the main complexities and how we addressed them in the manuscript below:

i) the models described both individual-level and population-level dynamics

To improve clarity, we presented individual-level followed by population-level methods and results in the respective sections systematically.

Methods: We added further explanation to Figure 1: “These flow diagrams describe within-host changes in resistant (R) and sensitive bacteria (S or s) carried by each individual patient in response to antibiotic consumption, bacterial growth and transmission events.” This was followed by Figure 2, which explained transitions in colonising bacteria in the overall ward population.

Results: We began by explaining an illustrative example (Figure 3) of how prolonging antibiotic duration affects the amount of susceptible and resistant bacteria carried within each individual patient (left sided graphs) and in the overall ward population (right sided graphs).

ii) a large number of parameters were included in three different models

Figure 4 presented a comprehensive list of parameters explored in all three models, grouped according to the main processes they describe. In the main text, we identified the most important parameters which include between-host transmission, and growth rate of bacteria in the gut. These parameters were then discussed in detail in subsequent paragraphs.

- **Discussion**

**Major comment: It should be mentioned as a limitation that both antibiotic use and resistance is much more complex than modelled here (many different antibiotics often in combination for different durations and routes of administrations and dosages over multiple courses with multiple different resistance pathogens with different resistance mechanisms)**

Thank you for this insightful comment. We added this to the discussion: “emergence and spread of antimicrobial resistance under antibiotic selective pressure are complex and cannot be fully described with any of the models presented. It is reassuring, however, that all three versions of the models produced similar conclusions.”

**Minor comment: "The study by D'Agata et al" This study seems to be missing from the references, idem Blanquart et al. It seems to me that there are two modelling papers by D'Agata looking at treatment duration and resistance (interestingly with somewhat opposing conclusions)**

**https://ddec1-0-en-ctp.trendmicro.com:443/wis/clicktime/v1/query?url=https%3a%2f%2fjournals.plos.org%2fplosone%2farticle%3fid%3d10.1371%2fjournal.pone.0004036&umid=faa8deae-c687-48ec-8691-5be8be7332aa&auth=8d3ccd473d52f326e51c0f75cb32c9541898e5d5-f99f32888f49f12f97d332279a11e7cd04a7b542**

[**https://ddec1-0-en-ctp.trendmicro.com:443/wis/clicktime/v1/query?url=https%3a%2f%2fwww.ncbi.nlm.nih.gov%2fpmc%2farticles%2fPMC2432019%2f&umid=faa8deae-c687-48ec-8691-5be8be7332aa&auth=8d3ccd473d52f326e51c0f75cb32c9541898e5d5-a9a3bc2692a6b9ca6b06cff961515494d70aaaef**](https://ddec1-0-en-ctp.trendmicro.com:443/wis/clicktime/v1/query?url=https%3a%2f%2fwww.ncbi.nlm.nih.gov%2fpmc%2farticles%2fPMC2432019%2f&umid=faa8deae-c687-48ec-8691-5be8be7332aa&auth=8d3ccd473d52f326e51c0f75cb32c9541898e5d5-a9a3bc2692a6b9ca6b06cff961515494d70aaaef)

Thank you for pointing this out. We have added the missing references, and added the additional reference as suggested in the discussion: “The first study by D’Agata et al is a theoretical agent-based model which concluded that rapid initiation of treatment and minimising duration were likely to reduce antimicrobial resistance in an hospital epidemic setting. *The second study by D’Agata et al focused on within-host dynamics of resistance by effective and ineffective antibiotic killing. It found that shorter and early interruption of antibiotic therapy selected resistant strains when antibiotic killing was effective.* ”

- **Figures and tables**

**Minor comment: I found figure 1 not simple to understand. Care should be taken to consider readability. The legends of some of the other figures were also difficult to follow (again I acknowledge that this is partly a consequence of the complexity of the topic and models).**

As explained above, we added further explanation to Figure 1: “These flow diagrams describe within-host changes in resistant (R) and sensitive bacteria (S or s) carried by each individual patient in response to antibiotic consumption, bacterial growth and transmission events.”

- **References**

**Minor comments: Some references are not formatted.**

Thank you for this comment. We have reformatted the references to the Plos Medicine requirements.

**Minor comment: There are now also some studies using metagenomics, that show that the impact of treatment duration on intestinal resistance genes for examples is far from obvious https://ddec1-0-en-ctp.trendmicro.com:443/wis/clicktime/v1/query?url=https%3a%2f%2fpubmed.ncbi.nlm.nih.gov%2f34492446%2f&umid=faa8deae-c687-48ec-8691-5be8be7332aa&auth=8d3ccd473d52f326e51c0f75cb32c9541898e5d5-c4bed8e777506c25f0973a070dba2ce00b3bddc1**

Thank you for this suggestion. We have included this new reference in the manuscript here: “However, while antibiotic selective pressure as a driver of resistance is not in doubt, there are many gaps in our understanding of the relationship between duration and the prevalence of resistance.”

**Comments from reviewer 2 (methodological reviewer):**

**"The effect of reducing antibiotic treatment duration on antimicrobial resistance in the healthcare setting: a modelling study and meta-analysis" examines said effect in two largely-separate ways: firstly, through mathematical modelling, and secondly through a meta-analysis, which yielded five suitable prior randomised trials. Both methods suggest that reduced antibiotic treatment duration can result in some reduction in resistance carriage. These findings appear plausible, although there are some reservations relating to the representativeness of the mathematical models, and the relatively small sample size of the meta-analysis. Detailed comments follow:**

**1. It might be clarified as to whether the three models presented (Figure 1) are standard/known from prior work, since there appears extant literature on mathematical/computational modelling for colonisation (resistance), e.g. Boureau, L. Hartmann, T. Karjalainen, I. Rowland, MHF Wilkinson, H. (2000). Models to study colonisation and colonisation resistance. Microbial Ecology in Health and Disease, 12(2), 247-258.**

We thank the reviewer for sharing with us this comprehensive review. The exclusive colonisation model adopts a commonly used structure where individuals could only assume a single carriage state of either colonisation by resistant or susceptible bacteria. We have added a reference for this model (Austin 1997). The second model, the co-colonisation model, allows for individuals to carry varying amounts of both resistant and susceptible bacteria. This model structure has also been previously used (Davies 2019). Lastly, in the within-host growth model, we used the logistic growth function similar to the Monod equation for growth. This generalised logistic growth equation allowed for intra and interspecies competition. We added the above suggested reference to this last model in the main manuscript. In all these models, the unique features were that we incorporated between-host transmission events, and assessed resistance carriers at both individual and population levels.

**2. In Supplementary S1.2, it is observed that many of the parameters do not appear to have relevant references cited in support of their chosen value ranges. For these parameters, the chosen ranges might be briefly justified.**

As earlier explained, for those parameter values not found in the literature, we explored the largest reasonable range of parameter values. We have added further footnotes to the table including: “Parameter ranges in terms of proportions or daily probabilities which are not supported by references were chosen using the largest sensible ranges.” Examples of these are probability of carrying resistant bacteria on admission, mean proportion reduction in susceptible bacteria due to effective antibiotic killing, and probability of being prescribed any antibiotics on admission day on. Ranges for these parameters were assigned typically from very small e.g. 0.001 to fairly large values e.g. 1.

**3. In Supplementary S1.3.1, Figure S2 appears to be labeled as S1. This might be addressed, together with its corresponding reference(s) in the text.**

Thank you for spotting this labelling error. We have made the corrections accordingly.

**4. In S1.3.2, it is stated that "Non-monotonicities in the correlations between the parameters and the model output were examined visually through scatter plots and calculating the Hoeffding's D measure and Spearman's rank correlation measure"; were any such non-monotonicities found, addressed further?**

Thank you for this comment. We have added “There were no non-monotonicities found within the explored parameter space.” in section S1.3.2.

**5. Figure S3 is stated to show scatter plots of the parameters as sampled from hypercubes formed within each parameter space. Then, it is assumed that for each model output (resistance/non-resistance carrier value), each point within the scatterplot for some input variable (e.g. n) represents the estimated relationship between that input variable and the output, with the remaining input variables values being randomly sampled. It is further assumed that each point derives from the "averaged model outputs from [100 iterations] for the same set of parameter combination inputs", as described in S.1.3.1.**

**If the above understanding is correct, it might be justified as to whether the 500 points (i.e. independent sets of [different] parameter combination inputs, from Line 190) that were used in the scatterplots and associated regression estimates is sufficient. This is because the number of (input) parameter combinations increases exponentially with the number of parameter (i.e. the "curse of dimensionality"). For example, if we consider that a parameter is merely binary (i.e. has a high or low value only), and there are 16 such parameters (as in the smallest exclusive colonisation model), then simply representing all binary input combinations would take 2^16=65,536 points.**

**An empirical method of testing this might be to re-run the experiments with a different seed/set of 500 unique sets of parameters), and to observe whether similar conclusions are obtained (as presented in Figure 4). This might be warranted as the effects appear minimal for most variables, although there does seem a consistent pattern for p_R on resistant carriers in overall ward population across all three models for example (which however seems somewhat tautological).**

We thank the reviewer for this astute comment. We re-ran all the models with 10 repeats of 500 unique sets of parameters and produced similar findings. Code for re-running these experiments are publicly available in the github repository. We have added in the caption for S3: “Similar graphs were produced using 10 other sets of 500 unique sets of parameters.”

**6. The caption of Figure S3 further states that the lines in the scatterplots are regression lines obtained from locally estimated scatterplot smoothing. The algorithm/procedure for plotting these lines from the points might be briefly described.**

Thank you for raising this query. The lines were plotted using loess (local regression fitting). We added this explanation to the graph caption: “The lines are regression lines obtained from locally estimated scatterplot smoothing (*local regression fitting*).”

**7. Tradeoffs associated with shortening antibiotic treatment duration might also be acknowledged and discussed, as otherwise a reduction to zero duration would appear optimal.**

We agree with the reviewer on this point. We have added in the introduction: “This includes *optimising* definitive antibiotic courses for established bacterial infections *while ensuring clinical cure*, and rapid discontinuation of empiric prescriptions after bacterial infections are ruled out. ”

**Minor issues:**

**(Line 78) "This selective pressure for resistance most obviously applies..." might be considered to be rephrased (perhaps as "most directly applies")**

Thank you for this suggestion. We have made the change: “This selective pressure for resistance most *clearly* applies to pathogens directly targeted by the antibiotic when used to treat or prevent infections.”.

**(Line 270) "then acted as a reservoirs" might be "reservior"**

Thank you for spotting this spelling error. We have made the suggested correction.

**Comments from reviewer 3:**

**Many thanks for the opportunity to review this interesting and well written manuscript by Mo and colleagues. The manuscript aimed to explore the relationship between antimicrobial duration and colonisation with resistant Gram-negative bacteria in a hospital setting. They aimed to compare the findings from reported models to observations reported in the literature by performing a systematic review and meta-analysis.**

**This study highlights the challenges of trying to determine the impact of any single intervention (in this case shortening treatment duration) on carriage / propagation of AMR. It approaches this in a logical and open way, acknowledging the limitations of the models used and attempting to triangulate observations where possible. It also reinforces many of the challenges we have in design and assessment of impact of stewardship programmes on AMR. A critical point is who, how often, and where we should be following up patients in trials to try and determine the true impact of reducing antimicrobial consumption on AMR (at the patient, ward, even regional level).**

**In terms of the models used and moving forwards, it would be interesting to understand whether these could now be applied to a population level, where we often consider antimicrobial consumption and the impact on AMR. For example, modelling a hospital using individual ward data predictions to highlight wards that are safe for stewardship interventions / focus on trying to reduce antimicrobial prescribing. Could it also better inform optimal times for sampling in these studies?**

We thank the reviewer for the encouraging comments. All three versions of the models incorporated between host transmission events such that the findings could be applied at a population level. Indeed, we found that infection prevention and control measures which reduce transmission events e.g. hand hygiene and environmental cleaning, are complementary to antibiotic stewardship policies which shorten duration.

To inform optimal times for sampling in studies which assess resistance carriage after implementation of an antibiotic prescription policy, a separate model will be required. Our models assumed that all patients in the ward were sampled daily.

**For the systematic review, the methodology is a little light.**

Thank you for this comment. Since this manuscript consists of both mathematical modelling (three versions of a similar model) and a systematic review, we had to shift some methodology details to the supplementary material. Supplementary material 2 contains the review protocol, search strings, PRISMA, quality assessment and meta-analysis models.

**Was the manuscript registered on prospero or similar prospective registry prior to starting?**

As explained above, we attempted to register the systematic review on PROSPERO. However due to the COVID-19 pandemic, COVID-19 relevant reviews were prioritised and the approval was delayed. We decided not to wait for this approval before commencing on the review.

**Was any assessment for risk of bias performed? Were the PRISMA reporting guidelines followed for systematic review and meta-analysis?**

Thank you for this comment. We have added risk of bias assessment and the PRISMA reporting checklist in the amended manuscript.

**Comments from reviewer 4:**

**L38 state the duration / duration reduced**

We thank the reviewer for this comment. We added the specific range of short and long durations used in the models in the methods section: “Exact duration for each antibiotic course was drawn from a uniform distribution (range for short duration 3 to 7 days, range for long duration 14 to 21 days).”. In our paper, we focus on Gram-negative bacilli infections and the associated resistance mechanisms. These infections may affect various organ systems with different disease severity. Hence, we are not able to recommend specific duration reduction to prevent emergence of resistance.

**L41 most effective… compared to…**

We have made this suggested change: “The models showed that shortening duration is most effective at reducing resistance carriage in high *compared to low* transmission settings.”.

**L43 “Importantly, shortening antibiotic treatment may increase resistance carriage when antibiotics are effective against resistant bacteria.” Unclear**

We have made the following change: “Importantly, shortening antibiotic treatment may increase resistance carriage when antibiotics *administered can effectively suppress the colonising bacteria with a particular resistance phenotype*.”

**L61 …of antibiotic stewardship programmes (ASP) – add reference**

Thank you for this suggestion. We have added appropriate references, including the US guideline from Infectious Diseases Society of America and the Society for Healthcare Epidemiology of America, and the WHO practical toolkit for antimicrobial stewardship.

**L62 do you mean shortening duration of empiric prescription, and discontinue / de-escalate when infection is confirmed or ruled out?**

Thank you for this comment. We refer to all situations where antibiotics prescribed are stopped including both empiric and definitive antibiotics. We have added the following: “This includes optimising *definitive antibiotic* courses for established bacterial infections, and rapid discontinuation of *empiric* prescriptions after bacterial infections are ruled out. ”.

**L71 add reference**

Thank you for this suggestion. We have added a reference: “Spellberg B, Rice LB. Duration of Antibiotic Therapy: Shorter Is Better. Ann Intern Med. 2019 Aug 6;171(3):210-211”.

**L75 instead of Table 1 write a few sentences / summary of these references**

Thank you for this suggestion. Table 1 is included in the results section as it contains the findings from our systematic review. A summary of these references are hence provided in the results section.

**L97 “from a systematic review…” should be you conducted a review? It sounds like you compared to another published review**

Thank you for this comment. We have made the following change: “We then sought to compare the model findings with empirical observations *by performing* a systematic review and meta-analysis.”

**L100 need one sentence to introduce the modelling method – what is a stochastic model, and what is agent-based, who / what is the agent in this study**

We have added the following to improve clarity:

i) In all models, we simulated individual patients *(agents)* in a single hospital ward environment.

ii) Because the population size under consideration is small and local fade-out events (when resistant populations reach zero) are potentially important, we considered stochastic implementations of these models (Figure 2, Supplementary material 1 section 1.1), *i.e. changes in colonisation status took place with a probability randomly drawn from a distribution.*

**L102 you did not provide the assumptions / model boundaries**

The model assumptions were explained with Figure 1 and subsections “Modelling dynamics of colonising bacteria in response to antibiotic treatment” in the methods section. Boundaries for the parameter values are included in Supplementary material 1 Table 1.2.

**L102 software packages and data sources???**

We have included the software packages in the methods section as follows: “All simulations were performed with R version 4.0.4 (2021-02-15) using packages msm, pse, and spartan.”

**L102 simulated for how long? I can see 60 days from the figures but need to state that clearly**

Thank you for this comment. We have added the following: “In all results shown below, each simulation was produced from at least 50,000 iterations (100 repeats of 500 unique sets of parameters selected within the ranges shown in Supplementary material 1 Table 1.2) *over 300 days*.” We optimised the number of days in the simulations by discarding the first 150 days to ensure equilibrium of the model is reached prior to calculating the model outputs (Supplementary material 1 Figure S1).

**L127 Figure 1 – need clear description of y and x axis, especially what do you mean by bed number, do you mean the number of patients you simulated in the ward – do you assume no ward traffic?**

Thank you for this comment. We have changed the y-axis label “Bed Number” to “Unique patients” and updated the y-axis tick labels to 1 to 10 indicating that 10 unique patients were in the simulated ward. New patients admitted to the ward were indicated by blue vertical lines in the top two rows of graphs. This is explained in the graph caption: “Vertical blue lines represent new admissions to the ward.”

**L132 – 161 assumptions and description of the figure should come before the figure otherwise confusing / difficult to read – especially assumptions**

Thank you for this suggestion. We have moved the figure captions to before the graphs.

**L132 do you consider all patients have the same carrier capacity of bacteria and gut flora does not change?**

We assumed a different carrier capacity for each individual in each iteration. The mean total carrying capacity for bacteria was drawn from a normal distribution on a log scale. This is explained in supplementary material 1 section S1.1. To clarify this in the main text, we added: “The mean total carrying capacity for each iteration was drawn from a normal distribution on a log scale.” in the main text.

**L212 search strings?**

Search strings are provided in Supplementary material 2 Table S2.2. We added this in the methodology section.

**L309 why not use a tornado plot, or even a feedback look?**

We thank the reviewer for this suggestion. We chose a heatmap over a tornado plot so that the partial rank correlation coefficient values can be compared across the two scenarios and three models (rows), as well as within each scenario and model (columns).

**L421 unclear whether the meta-analysis is a random or fixed effect model? I can see the authors tried to reduce the heterogeneity of the included studies but still need to consider how the results should be synthesized**

Thank you for raising this point of confusion. The meta-analysis model is a fixed effect model where each arm in each trial was assumed to be independent. We have added this in the methodology section: “The regression model assumed each arm in each trial to be independent (*fixed effect*)”

**L421 I cannot see what is statistically significant**

Thank you for this comment. We used a Bayesian model to estimate the change in the daily risk of acquiring resistant bacteria colonisation per day of antibiotic consumption and reported the 80% credible interval (7% absolute increase in risk of resistance carriage, 80% credible interval 3 to 11%). This interval can be interpreted with the readers’ judgement and clinical context in terms of significance. In addition, in line with the ASA consensus statement on p-values (<https://doi.org/10.1080/00031305.2016.1154108>) we don’t think that it is usually a good idea to dichotomise results into significant/non-significant.

**The authors did not consider the recommended duration of different types of antibiotics, and most importantly, are you only talking about intravenous? This is the major flaw / limitation.**

As reviewer 1 commented, it is a limitation for this work “that both antibiotic use and resistance is much more complex than modelled here (many different antibiotics often in combination for different durations and routes of administrations and dosages over multiple courses with multiple different resistance pathogens with different resistance mechanisms)”. Hence, we added this to the discussion: “emergence and spread of antimicrobial resistance under antibiotic selective pressure are complex and cannot be fully described with any of the models presented. It is reassuring, however, that all three versions of the models produced similar conclusions.” To clarify that we refer to both intravenous and oral antibiotics as systemic antibiotic treatment, we added: “Within the same ward, antibiotics (*administered via both intravenous and oral routes*) were prescribed with the same mean duration on or during admission.”

**Overall, this is valuable piece, agent-based modelling has been widely used to model AMR but modelling the impact of antibiotic duration has been limited.**

**However major revision is needed to 1) clearly state the model assumptions and data sources – the model boundary, the assumptions in bacteria growth, transmission route, host carrier capacity, type of treatment, and whether it is only empiric?**

**In real life it is very unrealistic to have some inpatient on empiric treatment for longer than 3 days without micro revision, this restricted the value of this study.**

Thank you for this comment. We hope with the above clarifications, most concerns have been addressed. Model boundary, assumptions and transmissions, host carrier capacity and types of antibiotics were detailed in the Supplementary material 1, and now added onto the main text as suggested. As for empiric versus definitive antibiotics, we focused on two types of antibiotics, one which is effective and one which is ineffective against the resistance phenotype. These may include both empiric and definitive antibiotics.

Once again, we sincerely thank you for your detailed comments and we forward to further correspondence.

Yours sincerely,

Mo Yin

MBBS, DPhil
