# Supplementary material for: Implications of reducing antibiotic treatment duration for antimicrobial resistance in hospital settings: A modelling study and meta-analysis
Source: PLoS Med. 2023 Jun 15;20(6):e1004013. doi: 10.1371/journal.pmed.1004013 (PMC10270346; doi:10.1371/journal.pmed.1004013)
Supplement: Attachment — Submitted filename: abm_abxdur_PlosMed_reply2.docx [file pmed.1004013.s005.docx]

Dr Philippa Dodd

Senior Editor

*PLoS Medicine*

3 September 2022

Dear Dr Dodd,

**Re: Manuscript revision: “Implications of reducing antibiotic treatment duration for antimicrobial resistance in hospital settings: A modelling study and meta-analysis” for PLoS Medicine**

Thank you very much for the reviewers’ and the editorial board’s acceptance of our manuscript. Please find the accompanying revised manuscript for your review and consideration. Our responses to the comments are as follows.

**Comments from Editors**

**Throughout, please ensure consistent use of terminology (e.g. antibiotic Vs agent)**

Thank you for pointing out this inconsistency. We have ensured “antibiotic” was used throughout the manuscript.

**Throughout, please ensure consistent use of words (five) or numbers (5) when presenting/discussing numerical values**

Thank you for pointing out this inconsistency. We have ensured numbers were used throughout the manuscript.

- **Abstract**

**Thank you for adding the following sentence to the abstract “The meta-analysis was performed with a logistic regression ….” Perhaps reword for improved clarity, suggest “The meta-analysis was performed using logistic regression. Duration of antibiotic treatment and time from administration of antibiotics to surveillance culture were included as independent variables” or something similar, as we understand it.**

Thank you for this suggestion. We have made this change.

**Thank you for adding the following sentence, “Interpretation of these estimates is limited by the low number of antibiotic duration trials which monitored resistance carriage as an outcome, contributing to a large credible interval.” Suggest modifying to “…which monitored carriage of resistant gram negative bacteria, as an outcome…” for clarity and consistency.**

Thank you for this suggestion. We have made this change.

- **Author Summary**

**Thank you for including an author summary. Please ensure consistent terminology is used throughout – suggest either antibiotic treatment or antibiotic duration or antibiotic treatment duration but not different terms, similarly for antibiotic Vs agent.**

Thank you for pointing out these inconsistencies. We have ensured “antibiotic” and “antibiotic treatment duration” were used.

**Some further suggestions are below:**

**Why was this study done?: Suggest revising the following sentence to “…this depends on the agent’s spectrum of coverage, pharmacokinetic and pharmacodynamic properties…” – as bioavailability forms part of the aforementioned.**

We have deleted “, and bioavailability where the bacteria are located” as advised.

**Consider revising the sentence beginning “Empirical evidence…” to “Empirical evidence and an understanding of the mechanisms by which antibiotic treatment duration effects the emergence and spread of antimicrobial resistance are lacking.” or something similar**

Thank you for this suggestion. We have made this change.

*** “…factors driving the effect [of] antibiotic…”? consider also adding the words “…[help to] inform…”**

Thank you for this suggestion. We have made these changes.

**What did the researchers do and find?: Some of these statements appear repetitive. Please refine this section to include the studies primary findings.**

We have clarified this section to include the study’s primary findings.

**Please add [antibiotic treatment] or something similar to the following sentence “The models showed that shortening [ ] duration is most effective at reducing resistance carriage in high transmission settings”**

Thank you for this suggestion. We have made this change.

**What do these findings mean?: Please revise this section and include two or three main implications of the study findings that you list above.**

**Some of the points currently in this section might better placed above - some more specific comments are below:**

**“Shortening antibiotic duration…” consider revising to “…Shortening the duration of antibiotic treatment may increase or decrease colonization by resistant bacteria depending upon the effectiveness of antibiotics” or something similar.**

Thank you for this comment. We have changed the sentence to “Shortening antibiotic treatment duration may increase or decrease colonisation by resistant bacteria depending upon the effectiveness of antibiotics at eliminating the resistant bacteria.”

**Please clarify the following statement “For treated individuals, shortening duration is most effective when resistant bacteria grow rapidly under antibiotic selection pressure and decline rapidly when stopping treatment.” Most effective at reducing resistance?**

Thank you for this suggestion. We have added the suggested text: “For treated individuals, the models showed that shortening antibiotic treatment duration is most effective at reducing resistance carriage when resistant bacteria grow rapidly under antibiotic selection pressure and decline rapidly when stopping treatment.”

**The following statement - “The modest effect of shortening treatment duration on resistant bacteria colonisation can be partially explained by typically short hospitalisation stays and a slow rate of decolonisation of resistant bacteria in patients previously exposed to antibiotics” is long and difficult to read/understand. Please either revise to improve accessibility to the reader or remove**

Thank you for this comment. We have shortened the sentence to “The modest effect of shortening antibiotic treatment duration on resistant bacteria colonisation can be partially explained by typically short hospitalisation and a slow rate of decolonisation of resistant bacteria.”

**Consider the following sentence, or something similar in place of the current “We found that an important interplay exists between shortening antibiotic treatment and reducing the transmission of resistant bacteria through infection prevention and control” as we understand it**

We have changed the sentence as advised to “We found that an important interaction exists between shortening antibiotic treatment and reducing the transmission of resistant bacteria through infection prevention and control.”

**Please remove the statement on study limitations**

Thank you for this suggestion. We have removed the statement on study limitations.

- **Methods & Findings**

**Thank you for including assessment of the individual risk of bias for each study included in the meta-analysis.**

**The assessment of publication bias doesn’t appear to be included. While it is good practice to include a minimum of 10 studies when assessing risk of bias using tests for funnel plot asymmetry (if this underpins the omission?), alternative strategies, such as Tang’s regression test (PMID: 29663281) could be an alternative approach in respect of your meta-analysis which includes 5 studies. You may find further guidance here PMID: 10812319 helpful also. An additional comment on the limitations of assessment of publication bias in your study (perhaps in the discussion section of the main manuscript) as appropriate, would also be beneficial.**

Thank you for this helpful suggestion. We have included a funnel plot to assess publication bias in Supplementary material 2 Figure S2.9. In this funnel plot, the absolute difference between the proportion of resistance carriers in those who received long versus short antibiotic treatment duration (x-axis) is plotted against the square root sample size (y-axis). However, it should be noted that in these 5 trials, antibiotic treatment days in the short and long antibiotic treatment arms differ. Hence, while this plot gives an idea of antibiotic treatment effect on resistance carriage in these 5 publications, the crude absolute difference in proportion of resistance carriers cannot be compared directly without accounting for the antibiotic treatment days. This underpins the rationale behind our meta-analysis, which used antibiotic treatment days in each randomisation arm as an independent variable. We explain these in the figure caption, rather than adding to the discussion section of the main manuscript.

- **Figures**

**Thank you for your attention to our requests regarding the figures.**

**It appears that throughout, the figures are now rather out of sink with the figure captions. For example line 162, Figure 1…the figure is below the figure caption. The same is seen with all subsequent figures in the main manuscript. Throughout, please place the figures above the figure caption and ensure that captions are clearly distinct from the main text.**

Thank you for this comment. We initially placed the captions above the figures according to an earlier suggestion by one of the reviewers. In this current version, we have moved the figures to be above the captions. The captions are now single-spaced to distinguish them from the main text.

**Thank you for altering the colour schemes in figure 1. It might be advisable to opt for colours that are very distant from red and green - the pink shading used in figure 1 appears rather red, perhaps varying shades of light and dark blue, for example, or something similar. In addition it is difficult to read the white letters overlying the yellow background so suggest amending for improved readability.**

Thank you for this comment! The shades of blue and pink were chosen from a colour-blind friendly palette by IBM:

(<https://davidmathlogic.com/colorblind/#%23D81B60-%231E88E5-%23FFC107-%23004D40>)

We have changed the colour of ‘s’ from white to dark grey in the light yellow boxes to improve visibility.

**Figures 2 and 3 (heatmaps) are unchanged in colour – perhaps because re-analysis may be required? If they can be changed then please do so otherwise please indicate in your response if and where colour schemes cannot be altered for any reason so that I cease to pester you about it!**

In both Figures 2 and 3, the green/teal and orange shades were chosen from a colour-blindness palette by Wong:

(Bang Wong, Nature. 2011 <https://www.nature.com/articles/nmeth.1618>)

The blue and pink coloured lines in the last panel were chosen from the IBM colour-blindness friendly palette as explained above.

**Throughout the manuscript, if/when figure colors are altered, please also remember to amend the descriptive text e.g. line 387 in reference to figure 4 “Red, blue and grey coloured rectangles…”**

Thank you for spotting these errors. We have changed this to “Pink, teal and grey coloured rectangles…”

- **References**

**Ref #9 please spell out the names of the first 6 authors names**

We have included the names of the first 6 authors for reference #9.

**Please ensure all web references have an access date e.g. ref #2**

We have included the access date for reference #2.

**Comments from Reviewers**

- **Reviewer #1**

**The authors have addressed all my major comments. I congratulate them for this important work.**

- **Reviewer #3**

**Authors have addressed my previous comments.**

**No additional comments to add.**

**Many thanks and congratulations.**

- **Reviewer #4**

**The authors have addressed the comments I have provided earlier and improved the overall quality of the manuscript significantly. The methodology section has been clarified, especially around the assumptions made to conceptualise the agent based model. The steps taken to simulate the model have now been clearly described to allow audience who were unfamiliar with the method to understand the approach. I believe the revised manuscript fits for publication and have no further comments.**

We thank all the reviewers for their insightful comments! We specifically address reviewer #2’s comments in the section below.

- **Reviewer #2 (methodological reviewer)**

**We thank the authors for largely addressing our previous comments. A few minor suggestions might be considered:**

**1. While 10 repeats for various parameter sets were attempted, the concern about the curse of dimensionality appears to still possibly apply to unexplored combinations of parameters. This might be briefly mentioned as a potential limitation.**

We thank the reviewer for this comment. We have added this as a limitation in Supplementary material 1 S1.3.2 Scatter plots: “A limitation here is that not all combinations of parameters could be explored.”

**2. For the loess, the relevant hyperparameters used for fitting might be included, if relevant.**

Thank you for this comment. We used a span of 0.75 (the default of LOESS in R which provides in most cases reasonable fits). We have added this in the caption for Supplementary material 1 Figure S3.

**3. While tradeoffs for optimizing antibiotic treatment duration are now briefly discussed, the condition of "...optimising definitive antibiotic courses for established bacterial infections while ensuring clinical cure, and rapid discontinuation of empiric prescriptions after bacterial infections" appears fairly vague. Have any quantitative guidelines for the tradeoffs been considered?**

We thank the reviewer for this insightful comment! Optimising antibiotic treatment duration is indeed an area of intensive research in randomised trials and observational clinical studies. Quantifying the tradeoffs is challenging and highly context specific e.g. bacterial pathogen, site of infection, host immunity. Since discussing this in detail is beyond the scope of this manuscript, we did not make further additions.

Once again, we sincerely thank you for your detailed comments and we forward to further correspondence.

Yours sincerely,

Mo Yin

MBBS, DPhil
