# Supplementary material for: Implications of reducing antibiotic treatment duration for antimicrobial resistance in hospital settings: A modelling study and meta-analysis
Source: PLoS Med. 2023 Jun 15;20(6):e1004013. doi: 10.1371/journal.pmed.1004013 (PMC10270346; doi:10.1371/journal.pmed.1004013)
Supplement: Attachment — Submitted filename: abm_abxdur_PlosMed_reply3.docx [file pmed.1004013.s006.docx]

Dr Philippa Dodd

Senior Editor

*PLoS Medicine*

4 October 2022

Dear Dr Dodd,

**Re: Manuscript revision: “Implications of reducing antibiotic treatment duration for antimicrobial resistance in hospital settings: A modelling study and meta-analysis” for PLoS Medicine**

Thank you very much for the reviewers’ and the editorial board’s acceptance of our manuscript. Please find the accompanying revised manuscript for your review and consideration. Our responses to the comments are as follows.

- **ABSTRACT**

**Line 48:  “Importantly, shortening antibiotic treatment may increase resistance carriage when antibiotics administered can effectively suppress the colonising bacteria with a particular resistance phenotype….”, is rather confusing to me. I have suggested a revision, below, as per my understanding – please correct this if I am mistaken! “Importantly, under circumstances whereby administered antibiotics can suppress colonizing bacteria, shortening antibiotic treatment may increase the carriage of a particular resistance phenotype.”**

Thank you for this great suggestion! We have amended this as recommended.

**Please also amend the abstract in the manuscript submission form when you resubmit**

Thank you for the reminder. We have done so.

**Line 50-53: beginning we “…We identified…” suggest revise to the following as per my understanding:
“We identified 187 randomised trials which investigated antibiotic duration. Of these, 5 reported resistant Gram-negative bacteria carriage as an outcome and were included in the meta-analysis. The meta-analysis determined that a single additional antibiotic treatment day is associated with a 7% absolute increase in risk of resistance carriage (80% credible interval 3 to 11%).”**

Thank you for this edit! We have amended this as recommended.

**Notably, the information in the abstract and main manuscript differ:
Line 492: the section titled “evidence from randomized controlled trials” will need revising.**

**Line 493: “Initial search of the MEDLINE and EMBASE databases returned 2407 unique publications. Out of these 187 were randomised trials which compared antibiotic treatment durations. 7 these trials collected surveillance cultures for colonising bacteria during follow-up visits and were included in the qualitative synthesis.” However in the abstract report the inclusion of 5 studies….
Only later do we find out that a further 2 of these studies were excluded which is a bit confusing. For clarity and transparency the information from line 512 should be reported earlier, at least before we are directed to the table of study characteristics which reports 5 not 7 studies.**

We apologise for the confusion. 7 (now updated to 10) studies were discussed *qualitatively* in the paragraph starting from line 510. Out of which, 5 were included in the *quantitative* meta-analysis.

We have reported information from line 512 in the earlier paragraph starting from line 503, and referred to Table 1 following this sentence: “A meta-regression analysis was performed using 5 out of the 10 trials (Table 1).”

**Table 1 also only details 5 studies rather than 7…please also update the PRISMA flow charts for study exclusion as necessary

In the abstract, you report the search was performed up to May 2021, can you please update it to within the last 6 months.  I suspect there will not be any additional trials to include but please update the search dates in any case and the relevant PRISMA flowcharts etc.**

**Please include the updated search dates in the main manuscript text where you describe the metanalysis and systematic review (line 277). Please ensure any updates are also made to supplementary files where relevant**

We have updated the PRISMA diagram according to the above recommendations. Specifically, we indicated 2 studies were not included in the meta-analysis, and updated the search to 4 October 2022. We have also updated the text in the main manuscript and supplementary material 2 to reflect the latest search.

In this updated search, we found 3 more antibiotic duration randomised trials which performed surveillance cultures. However, all 3 were excluded from the meta-analysis due to the following reasons:

- 1 was excluded because the trial reported a mixture of antibiotics and multidrug-resistant bacteria carriage without specifying if the organisms were Gram-positive or Gram-negative.
- 2 were excluded because the study only collected Gram-positive bacteria in surveillance cultures
- **AUTHOR SUMMARY**

**This should be concise, distinct from the abstract and accessible to the lay reader.  I have revised, as per my understanding, as below:

Why was this study done?
• Shortening antibiotic treatment duration is a commonly adopted antibiotic stewardship strategy, with the expectation that it will reduce antimicrobial resistance in treated individuals and in the overall population.
• Antibiotic selective pressure acts predominantly on ‘bystander’ colonising bacteria for resistance, and this depends on the spectrum of coverage, pharmacokinetic and pharmacodynamic properties of individual antibiotics.
• Empirical evidence and an understanding of the mechanisms by which antibiotic treatment duration effects the emergence and spread of antimicrobial resistance are lacking. Understanding the key factors driving the effect of antibiotic treatment duration on resistance carriage will help to inform future research study designs, antimicrobial stewardship interventions and resource allocation in the multimodal control strategies.

What did the researchers do and find?
• We modelled within- and between-host dynamics of colonising ‘bystander’ susceptible and resistant bacteria in response to systemic antibiotic treatment and compared the model findings with a systematic review and meta-analysis.
• The meta-analysis found one additional antibiotic treatment day is associated with a 7% absolute increase in risk of resistance carriage when antibiotics administered were not effective against the resistance phenotype in the colonising bacteria.
• For treated individuals, the models showed that shortening antibiotic treatment duration is most effective at reducing resistance carriage when resistant bacteria grow rapidly under antibiotic selection pressure and decline rapidly when stopping treatment.
• At a population level, shortening antibiotic treatment duration is most effective at reducing resistance carriage in high transmission settings.
• Shortening antibiotic treatment duration may increase resistance carriage when the antibiotics administered are effective at eliminating colonising bacteria with a particular resistance phenotype.

What do these findings mean?
• Shortening antibiotic treatment duration may increase or decrease colonisation by resistant bacteria, dependent upon individual and combined bacterial and antibiotic characteristics, and may help to inform antimicrobial stewardship policy making.

Please amend if I am mistaken in my interpretation at any point in editing this summary**

Thank you for these suggestions! We have edited the first two sections as advised above. In the last section “What do these findings mean?”, we have changed it to the following:

- Shortening antibiotic treatment duration may increase or decrease colonisation by resistant bacteria, dependent upon individual and combined bacterial and antibiotic characteristics.
- The effect of shortening antibiotic treatment duration on colonisation by resistant bacteria colonisation is potentially modest due to short hospitalisation periods and slow decolonisation of resistant bacteria.
- These findings can inform antibiotic stewardship programmes to shorten antibiotic treatment and infection prevention and control policies to reduce transmission of resistant bacteria.
- **GENERIC INFORMATION FOR MODELLING STUDIES**

**Please check the list below and ensure details are adequately detailed in the manuscript

Please provide a diagram that shows the model structure, including how the disease natural history is represented, the process and determinants of disease acquisition, and how the putative intervention could affect the system.**

This is included in Figure 1.

**Please provide a complete list of model parameters, including clear and precise descriptions of [the meaning of each parameter, together with the values or ranges for each, with justification or the primary source cited, and important caveats about the use of these values noted].**

This is explained in the Methods section, and the corresponding table is in Supplementary material 1.

**Please provide a clear statement about how the model was fitted to the data [including goodness-of-fit measure, the numerical algorithm used, which parameter varied, constraints imposed on parameter values, and starting conditions].**

These are explained for both the agent based model and meta-analysis regression model in the methods and results sections. **For uncertainty analyses, please state the sources of uncertainties quantified and not quantified [can include parameter, data, and model structure].**

A comprehensive sensitivity analysis was performed for the agent based models to capture uncertainties in the relationship between antibiotic treatment duration and resistant bacteria carriage. We illustrate these uncertainties graphically in Figures 6 and 7 and throughout the Results section.

**Please provide sensitivity analyses to identify which parameter values are most important in the model. Uncertainty estimates seek to derive a range of credible results on the basis of an exploration of the range of reasonable parameter values. The choice of method should be presented and justified.**

The main work in this manuscript is a detailed sensitivity analysis to identify important parameters.

**Please discuss the scientific rationale for this choice of model structure and identify points where this choice could influence conclusions drawn. Please also describe the strength of the scientific basis underlying the key model assumptions.**

Agent based models were used to capture the complex intermediary steps between antibiotic consumption and resistant bacteria carriage. This is highlighted in the Methods section: “3 stochastic agent-based models with increasing complexity and biological realism were constructed.” In addition, we explained the rationale for a stochastic implementation: “Because the population size under consideration is small and local fade-out events (when resistant populations reach zero) are potentially important, we considered stochastic implementations of these models”.

- **REFERENCES**

**Line 569: “…community were used.[39]…” this error is repeated throughout the manuscript, please check and amend throughout as per below:
Please select the PLOS Medicine reference style in your citation manager. Please use square brackets for in-text reference call outs noting the absence of spaces within the square brackets, the space before the first bracket and the punctuation following the second bracket, “…symptomatic [2,8].”**

We have moved the punctuations to behind the references.

**PLOS uses the reference style outlined by the International Committee of Medical Journal Editors (ICMJE), also referred to as the “Vancouver” style. Example formats are listed below. Additional examples are in the ICMJE sample references.  Please list up to 6 author names only before et al where more than 8 authors have contributed

Example:        Hou WR, Hou YL, Wu GF, Song Y, Su XL, Sun B, et al. cDNA, genomic sequence cloning and overexpression of ribosomal protein gene L9 (rpL9) of the giant panda (Ailuropoda melanoleuca). Genet Mol Res. 2011;10: 1576-1588.**

We have edited the references to the above format.

Once again, we sincerely thank you for your detailed comments and we forward to further correspondence.

Yours sincerely,

Mo Yin

MBBS, DPhil
